# Colloidal Chemistry in Water Treatment: The Effect of Ca^2+^ on the Interaction between Humic Acid and Poly(diallyldimethylammonium chloride) (PDADMAC)

**DOI:** 10.1021/acs.langmuir.3c03029

**Published:** 2024-02-19

**Authors:** Mingyu Yuan, Heriberto Bustamante, Najet Mahmoudi, Michael Gradzielski

**Affiliations:** † Stranski-Laboratorium für Physikalische und Theoretische Chemie, Institut für Chemie, Technische Universität Berlin, D-10623 Berlin, Germany; ‡ Sydney Water, Parramatta, NSW 2125, Australia; § ISIS Neutron and Muon Source, Rutherford Appleton Laboratory, Harwell Oxford, Didcot OX11 0QX, United Kingdom

## Abstract

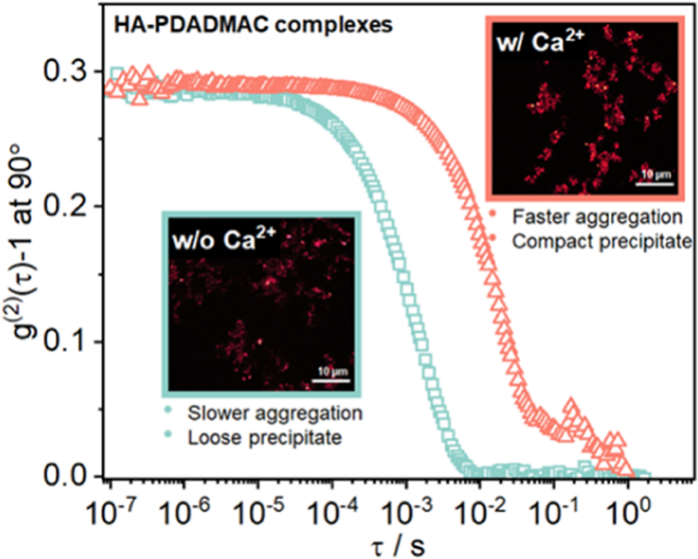

The complexation of humic acid (HA), as a major component of natural organic matter (NOM) in raw water, with polycations is a key step in the water treatment process. At sufficiently high addition of a polycation, it leads to neutralization of the formed complexes and precipitation. In this work, we studied the effect of the presence of Ca^2+^ ions on this process, with poly(diallyldimethylammonium chloride) (PDADMAC) as a polycation. This was done by determining the phase behavior and characterizing the structures in solution by light scattering and small-angle neutron scattering (SANS). We observe that with increasing Ca^2+^ concentration, the phase boundaries of the precipitation region shift to a lower PDADMAC concentration, which coincides well with a shift of the ζ-potential of the aggregates in solution. Light scattering shows the formation of aggregates of a 120–150 nm radius, and SANS shows that Ca^2+^ addition promotes a compaction in the size range of 10–50 nm within these aggregates. This agrees well with the observation of more densely packed precipitates by confocal microscopy in the presence of Ca^2+^. Following the precipitation kinetics by turbidimetry shows a marked speeding up of the process already in the presence of rather small Ca^2+^ concentrations of 1 mg/L. It can be stated that the presence of Ca^2+^ during the complexation process of HA with a polycation has a marked effect on phase behavior and precipitation kinetics of the formed aggregates. In general, the presence of Ca^2+^ facilitates the process largely already at rather low concentrations, and this appears to be linked to a compaction of the formed structures in the mesoscopic size range of about 10–50 nm. These findings should be of significant importance for tailoring the flocculation process in water treatment, which is a highly important process in delivering drinking water of sufficient quality to humans.

## Introduction

The production of clean drinking water is one of the main challenges for humanity. Normal surface water contains large amounts of natural organic matter (NOM) that arise from natural biological degradation of organic life substances and is mostly composed of humic substances, but may vary largely with respect to its detailed composition.^1^ NOM might partly be toxic but becomes even more so during the necessary disinfection process, in which conventionally chlorine (Cl_2_) is used as a disinfectant in the form of disinfection byproducts (DBPs), for instance, trihalomethanes (THM).^2^ Humic acid (HA), as the main component (∼60 wt %) of NOM,^3^ is a complex mixture of a large number of organic molecules with *M*_w_ ranging from 400 Da to 300 kDa.^4^ It is composed of a large variety of organic macromolecules with carboxylic and phenolic groups, which contribute mostly to the negative surface charge and reactivity of humic acid.^5^ Various approaches to remove NOM from drinking water have been reviewed recently.^6−8^

Traditionally, the main treatment in water purification is addition of an inorganic coagulant (Fe^III^ or Al^III^), which typically is followed by the addition of a polycation, such as poly(diallyldimethylammonium chloride) (PDADMAC).^8−10^ It is well established that by adding positively charged multivalent metal ions at a relatively low pH value, charge neutralization is achieved that reduces the repulsion between predominantly negatively charged HA molecules so that this colloidal system is destabilized enough to start coagulation. At higher pH values (usually the case in the practice of water treatment and achieved by lime addition, i.e., addition of Ca^2+^ ions), the hydrolysis of metal ions becomes the main reaction and NOM is removed by precipitation.^11^ However, such “sweep flocculation” may not be sufficient enough for practical usage as, for example, in some water treatment plants in Australia, only around 20–30% of the NOM can be removed,^12^ which necessitates additional use of polycations to remove the remaining soluble NOM that has not been removed by an inorganic coagulant (Fe^III^ or Al^III^) yet. When oppositely charged polymers (i.e., polycations) are added, coagulated particles and microflocs are brought together to form flocculates, which can be removed easily. The combination of these two steps is called coagulation–flocculation, which is widely used in water purification because of its high efficiency, simple operation, and low cost.^13,14^ It might be noted that there are methods like membrane filtration that can be used for water purification^15^ and one may also substitute chlorine with ozone^16^ or UV treatment^17^ for sterilization, thereby avoiding the risk of chlorinated DBP. However, at the current state, the coagulation–flocculation method, due to its ease of large-scale application, as well as associated low cost, still remains the most largely employed method for water treatment.

From a colloidal point of view, the interaction of a polycation and HA is the formation of a classical interpolyelectrolyte complex (IPEC)^18^ of oppositely charged polyelectrolytes, as known from the 1940s.^19^ In this process, HA can be regarded as a polyanion with high polydispersity, rich in aromatic groups, and rather rigid as a macromolecule.^20^ Polymer binding plays an important role in the complexation between HA and PE, which can be regarded to be largely irreversible as it is unlikely for all complexed segments in a long polymer chain to be detached simultaneously.^21^ Based on a mechanism recognized since the 1950s,^22^ when the polymer chain is long enough to project into solutions, these “dangling” polymer segments adsorbed on particles can “bridge” particles together to form strong flocculates. Several previous studies reported HA removal with PE as a function of PE dosage and pH level.^23−25^ Basically, the optimum HA removal happens when the PE dosage reaches the level required for charge neutralization, which is at a relatively high pH value, and the high ionization of HA molecules is favorable for complete complexation with PE. In addition, HA removal can be enhanced by PE with higher charge density, while the effect of molecular weight, *M*_w_, of PE (typically in the range of 50 kDa to 150 kDa) is negligible above a certain critical value of *M*_w_.^26^

In addition, the presence of metal cations may play an important role in the aggregation behavior of HA and also modulate its interaction with polyelectrolytes.^23^ The change of aggregate sizes of humic acid under various temperatures, cation conditions, and pH values was investigated by dynamic light scattering (DLS), observing particle diameters of 100–500 nm, depending on the origin of the HA.^27^ It was found that within an ambient range, a higher temperature leads to bigger humic acid aggregates, which can be related to temperature-induced clouding.^28^ Furthermore, acidification caused shrinking of HA aggregates as the repulsion of negatively charged sites was reduced. Upon further acidification, intermolecular aggregation was induced and eventually led to precipitation.

The complexation of HA with metal ions has been widely studied.^29^ For instance, potentiometric titrations have shown that the charge of humic acid becomes reduced by the presence of Ca^2+^, which can be attributed to binding of the Ca^2+^ ions to the carboxylic acid groups.^30^ Specifically, an initial decrease in the aggregate size of humic acid was found with addition of up to 0.5 mM Mg^2+^ ions to a 20 mg/L HA solution. This suggests charge neutralization and internal cross-linking of HA by added cations, which leads to intramolecular contraction and ultimately forms a more compact configuration. Following similar trends of HA aggregate size upon further acidification, the further addition of Mg^2+^ ions led to intermolecular aggregation. Note that intramolecular contraction was seen for both Mg^2+^ and Na^+^ but was pronounced with more highly charged cations, and only for Mg^2+^, intermolecular aggregation was seen, indicating the inability to induce intermolecular associations by monovalent cations.^27^ With trivalent ions, the HA aggregation was even further prompted as formation of pseudomicelles in aqueous humic acid was reported in the presence of Lu^3+^.^31^ Such comparison among various ions was also clarified by a molecular dynamic (MD) simulation study,^32^ which suggests that trivalent Eu^3+^ and bivalent Sr^2+^ may form inter/intramolecular bridges between negatively charged HA molecules to prompt coagulation, while for monovalent Cs^+^, no intermolecular bridges can be formed.

With higher concentrations of cations ranging from 0.001 to 0.5 M, the size of Suwannee River humic acid (SRHA) increases with Na^+^ concentration until a maximum is reached at 0.05 M, followed by a decrease for higher concentrations. While for HA in the presence of Ca^2+^, a consistent size increase was reported with increasing Ca^2+^ concentration up to 0.5 M, in which a marked increase in HA size happened even for Ca^2+^ concentrations lower than 0.02 M, which was followed by a less pronounced increase for higher Ca^2+^ concentrations in the range from 0.02 to 0.5 M.^33^ Apart from the investigation of aggregate size, the strength (against breakage) of HA aggregates was probed, as it plays a vital role in the removal process of HA in the water industry. Even in the presence of inorganic cations with the same valency, it was reported that the strength of HA aggregates was higher in the presence of Ca^2+^ than for Mg^2+^,^34^ which is consistent with higher efficiency of HA coagulation with a cation of a larger ionic radius and the same valency.^35^ Such a comparison was further confirmed by an MD simulation study, which clarified the monodentate and bidentate coordination between Ca^2+^ and carboxylate groups among the complexation of HA and cationic ions, while for Mg^2+^, only monodentate coordination was found.^36^

As Ca^2+^ ions are omnipresent in raw water and often become added in the form of lime during the water treatment process, a large number of studies have been dedicated to elucidating the Ca^2+^ effect within the conventional water treatment process. For instance, it has been found that the consumption of aluminum sulfate (Al_2_(SO4)_3_) was sufficiently reduced by around 20% to achieve comparable removal efficiency at pH above 6 when adopting CaCl_2_ as a cocoagulant to remove NOM from raw water.^37^ Even in Fe-HA systems, where the dosage of Fe was too low to form filterable HA flocs, the addition of Ca^2+^ increased the ζ-potential and enhanced the hydrolysis of Fe species, thereby improving HA removal.^38^ Interestingly, although Ca^2+^ is capable of compressing the electric double layers and neutralizing negatively charged HA molecules, the charge neutralization point cannot be reached only by addition of Ca^2+^ to HA, and its prompting effect for HA removal with polyaluminum chloride (PAC) is only observed when the amount of the coagulant is insufficient.^39^ However, the Ca^2+^ effect on the interaction between HA and cationic PE has been studied to a much lesser extent, despite the fact that it can also be expected to play an important role here, and the HA–PE interaction is an elementary step in the more complex process of water treatment.

In order to fill this knowledge gap, in our work, we focused on the effect of the presence of Ca^2+^ on the phase behavior and structures formed by humic acid (HA) in the presence of variable concentrations of high *M*_w_ (500 kDa) polycation PDADMAC at pH 9, where both carboxylic and phenolic groups are largely dissociated and high surface charge is achieved,^40^ which is also close to the experimentally prevalent conditions in the water treatment process. In order to have experimentally more well-defined and reproducible conditions, we worked with purified HA. The Ca^2+^ concentration varied from 0 to 0.25 mM, which corresponds well to the Ca^2+^ concentration seen in surface water that generally contains 1–2 mg/L (0.025–0.05 mM) but may reach 100 mg/L (2.5 mM) in lime areas.^41,42^ Not only the mesoscopic structure of HA-PDADMAC complexes with various concentrations of Ca^2+^ was probed by a combination of static and dynamic light scattering (SLS, DLS) and small-angle neutron scattering (SANS), but also the macroscopic sedimentation of HA flocs was monitored via long-time ultraviolet–visible (UV–vis) measurement. Finally, we also compared the effect of Ca^2+^ to that of other ions like Na^+^ and Mg^2+^ in order to discern effects that arise generically from the ionic strength and ion-specific effects of Ca^2+^.

## Experimental Methods

### Materials

Humic acid (HA) was purchased as a commercial product from Sigma-Aldrich (53680-50G, technical grade). It was further purified to remove fulvic acid (FA) and humin. For that purpose, raw HA was dissolved in a NaOH solution with pH over 12 and then filtered twice (LLG-Qualitative filter paper, pore size 5–13 μm and LABSOLUTE micro glass fiber filters, particle retention 1.20 μm). Then, 35% HCl was added to the filtered solution to lower the pH to 1 for precipitating HA. The obtained HA slurry was separated by centrifugation followed by freeze-drying to get a black HA powder. Further, 500 kDa poly(diallyldimethylammonium chloride) (PDADMAC) (high molecular weight, 400–500 kDa) was purchased from Sigma-Aldrich as 20 wt % solutions in water and freeze-dried before usage. A stock solution of PDADMAC was prepared at 0.01 wt %. Assuming each repeating unit of PDADMAC as a single charged unit (*M*_w_(charge) = 161.67 g/mol), this corresponds to a nominal charge concentration of 0.625 mM.

For all samples, except for SANS, the HA concentration was 40 mg/L, which corresponded to a nominal charge concentration of 0.135 mM, as calculated from the average molecular weight of the charged unit determined from titration (*M*_w_(charge) = 296 g/mol; see the titration curve in Figure S1). The pH of HA solutions was adjusted to 9.0 with 0.1 M NaOH solutions before the addition of PDADMAC. The PDADMAC concentration was varied to achieve a specific nominal charge ratio *Z*, which is defined as *Z* = [charges of PDADMAC]/[charges of HA]. Amounts of NaCl, MgCl_2_, and CaCl_2_ were added as calculated. All samples were prepared with Milli-Q water (18.2 MΩ·cm at 25 °C) or D_2_O (99.9% isotopic purity, Deutero GmBH) for SANS tests. All measurements were performed at least 24 h after sample preparation at 298 K.

#### Confocal Laser Scanning Microscopy (CLSM)

Confocal laser scanning microscopy imaging of precipitated flocs was performed with a Leica CLSM system in reflectance configuration using a 488 nm laser. Precipitated flocs were transferred from the bottom of a vial to a glass slide with a pipet 48 h after mixing. For better visualization, a lookup table (LUT) was applied to alter the color input values in the images for better visualization.

#### ζ-Potential

ζ-Potential measurements were performed using a Litesizer 500 instrument (Anton Paar) at 298 K and a wavelength of 658 nm with a laser power of 40 mW. ζ-potential was measured by electrophoretic light scattering (ELS), which measures the electrophoretic mobility *U*_E_ as
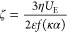
1where η is the dynamic viscosity, *U*_E_ is the electrophoretic mobility, ε is the dielectric constant, and *f*(κα) is the Debye factor, which was set to a value of 1.5 for particles suspended in aqueous solutions according to the Smoluchowski approximation.

#### UV Absorbance

UV absorbance of HA solutions was measured using a Cary 50 UV–vis spectrometer with a 1 cm quartz cell. In particular, we took the absorbance observed at 254 nm (known as UV254), and for calibration measured HA solutions at pH 9 in the concentration regime of 0.1–10 mg/L. From the linear relationship *y* = 0.04421*x* – 0.00694 (*R*^2^ = 0.9998) between UV254 and the HA concentration (*x* in mg/L), the amount of HA in unknown solutions can be determined reliably, and accordingly, the remaining HA can be quantitatively expressed as
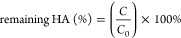
2where *C*_0_ is the original concentration of HA, typically 40 mg/L, and *C* is the concentration of HA remaining in the supernatant after precipitation, calculated from the UV–vis absorbance value at 254 nm.

#### Static and Dynamic Light Scattering (SLS and DLS)

Static and dynamic light scattering were measured simultaneously using an ALV/CGS-3 instrument. The instrument is equipped with a He–Ne laser with a wavelength λ of 632.8 nm. Samples were measured inside a temperature-controlled toluene bath, and measurements were done at 25 °C. Light scattering was recorded at several scattering angles θ, ranging from 30 to 130° set by an ALV-SP goniometer. The data were analyzed with in-house software called SimplightQt. The intensity autocorrelation function *g*^(2)^ (τ) was recorded via an ALV 5000/E multiple τ correlator from DLS measurements.

The SLS curves were recorded with the corresponding magnitude of the scattering vector *q*, with
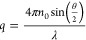
3where *n*_0_ is the refractive index of the solution and θ is the scattering angle.

The low-*q* region of the SLS curves, characteristic of the overall dimension of HA complexes, was analyzed with the Guinier approximation in order to obtain the forward scattering intensity at zero angle *I*_0_(*q* = 0) and the gyration radius *R*_g_
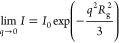
4The DLS intensity autocorrelation functions *g*^(2)^(τ) were fitted with a stretched exponential function to determine the relaxation rate Γ
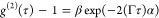
5where β is the intercept of the correlation function (for this instrument it should ideally be 0.333), α is the stretching exponent, and Γ is the relaxation rate.

From the slope of Γ versus *q*^2^, diffusion coefficient *D* can be calculated. With an assumption that the particles are spherical and noninteracting, the hydrodynamic radius *R*_h_ was derived from the Stokes–Einstein equation
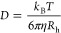
6where *k*_B_ is the Boltzmann constant, *T* is the absolute temperature, and η is the solvent viscosity

#### Small-Angle Neutron Scattering (SANS)

Small-angle neutron scattering experiments weere performed with the Sans2d time-of-flight SANS instrument at the ISIS Neutron and Muon Source (STFC, Rutherford Appleton Laboratory, U.K.). The experimental *q*-range covered was 0.0023–1 Å^–1^ with two detectors at 4 and 8 m from the sample, respectively, and the incident neutron wavelength ranging from 1.75 to 14.4 Å. Samples were measured in quartz cuvettes with an optical path length of 2 mm. *A* thermostatic sample changer was utilized for the experiment, and measurements were done at 25 °C. Data reduction was performed with Mantid,^43^ following the standard procedures for the instrument (detector efficiencies, measured sample transmissions, absolute scale using the scattering from a standard polymer, etc.)^44^ and data analysis was performed with SasView Version 5.0.5, an open-source scattering analysis software.^45^

The SANS results were fitted by a shape-independent empirical two-power law model, where the scattering intensity *I*(*q*) is given as
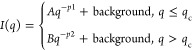
7where *q*_c_ is the location of the crossover from one slope to the other, *A* is a scaling coefficient that sets the overall intensity of the lower *q* power law region, and *p*1 and *p*2 are the power law exponents at low *q* and high *q*, respectively. The scaling of the second power law region (coefficient *B*) is then automatically scaled to match the first by the following formula
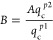
8During fitting, *A*, *q*_c_, *p*1, and *p*2 were free parameters.

As a second model, we also applied a shape-independent Beaucage model,^46−48^ which is generally very suited for describing structures with different levels or hierarchical organization and can reasonably approximate the scattering from many different types of particles, including fractal clusters, random coils, etc. The scattering intensity *I*(*q*) is given as
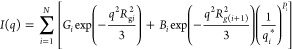
9
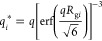
10where *G*_*i*_ is the scaling prefactor, *B*_*i*_ is the power law scattering prefactor, *R*_g_ is the radius of gyration, and *P*_*i*_ is the power law exponent. For a pure humic acid solution under various ionic conditions, a one-level Beaucage model (*N* = 1) was adopted; for the rest of the sample sets where PDADMAC is involved, the two-level Beaucage model (*N* = 2) was utilized in which *R*_g1_ refers to the radius of gyration at a larger scale and *R*_g2_ refers to the radius of gyration at a smaller scale, i,e, the local structure, respectively.

## Results and Discussion

The aim of this work was to elucidate in detail the phase behavior of complexes formed by humic acid (HA) and the polycation PDADMAC (500 kDa) and, in particular, how this process is affected by the presence of Ca^2+^ ions, which are automatically present in the process of water treatment under real conditions (e. g., the concentration of Ca^2+^ has an average value of 15 mg/L (0.375 mM) in the Nepean water treatment plant (WTP) in the Sydney area). Here, we were first interested in the changes in macroscopic phase behavior upon variation of Ca^2+^ concentration, as well as in the ensuing flocculation process. This was complemented by a thorough characterization of the structures of the soluble complexes, as well as of the precipitates formed. These are important aspects of the colloidal complexation process that are directly related to a central elementary step done in water treatment in the water industry, which is the interaction of humic acid with polycations. Therefore, this investigation is supposed to give fundamental insights into these elementary steps involved in the practical and more complex process of water treatment.

### Phase Behavior

As a first step, we characterized in detail the macroscopic phase behavior of aqueous solutions of humic acid containing different amounts of Ca^2+^ to which increasing amounts of PDADMAC were added, where the composition was described by the nominal charge ratio *Z*, which is defined as *Z* = [charges of PDADMAC]/[charges of HA]. It should be noted that these are always potential charges, ignoring possible protonation of the HA. These series contained different, constant concentrations of Ca^2+^, and we always worked with an HA concentration of 40 mg/L. Phase behavior of the mixed samples was visually examined 24 h after the preparation (Figure S1 in Supporting Information); this time interval was chosen to allow for adequate equilibration and the manifestation of any latent phase transitions.

For the reference HA-PDADMAC system without Ca^2+^ addition, the transition from the monophasic to the biphasic region can be observed at *Z* = 0.9, i.e., near the charge neutralization point arising from the complexation of negatively charged humic acid molecules by positively charged PDADMAC molecules. With vanishing electrical repulsion between the HA molecules, the system becomes destabilized, and flocs are formed and grow until finally, macroscopic phase separation takes place. As the dosage of PDADMAC is further increased to *Z* = 1.6, the positive excess charges from PDADMAC restabilize the system so that soluble complexes of humic acid are formed, switching back to a monophasic region, where a clear brownish solution with water-like viscosity is observed. In the biphasic regime, solutions become turbid upon mixing, and a dispersion of extremely fine flocs can be seen by the naked eye within ∼30 min. With the agglomeration of fine flocs and further sedimentation, phase separation occurs within several hours, resulting in a clear water-like supernatant and a dark-brown loosely packed precipitate of humic acid. The visual appearance of samples 24 h after mixing is shown by photos in Figure S2. It should be noted that samples were always prepared such that they avoided to pass through the equimolar charge regime (*Z* = 1) during the mixing process. An interesting point is that formed precipitates in the biphasic region cannot become dissolved again by further addition of PDADMAC, for instance, by moving the system to a *Z* value above 1.3 where the monophasic region is observed otherwise. Apparently, the formed precipitate is rather stable and its redispersion is kinetically hindered.

**Figure 1:**
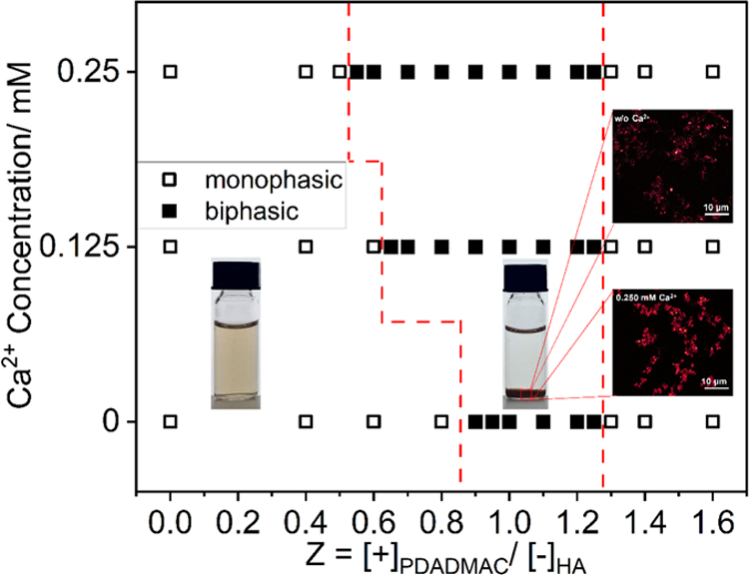
Phase diagram of 40 mg/L HA and added 500 kDa PDADMAC, the added amount being characterized by the nominal charge ratio *Z* (= [+]/[−]) for different concentrations of Ca^2+^ at pH 9.0. The inserts show confocal laser scanning microscopy (CLSM) images of precipitated flocs at Z = 1.0 with no Ca^2+^ and 0.250 mM Ca^2+^, respectively.

In comparison, when Ca^2+^ is present in the system, the range of precipitation is shifted to a much lower PDADMAC concentration (lower *Z*) for increasing Ca^2+^ concentration and this effect is already seen for rather low concentrations of 0.125 and 0.25 mM. This is shown in the phase diagram depicted in Figure 1, and one observes that for 0.125 mM Ca^2+^ and 0.25 mM Ca^2+^, the phase boundary appears at *Z* ∼ 0.62 and *Z* ∼ 0.52, respectively, while without Ca^2+^, it is at *Z* ∼ 0.85, i.e., much less PDADMAC is required to achieve precipitation of HA. It should be noted that the Ca^2+^ concentration here is directly in the range of the concentration of ionizable groups (i.e., carboxylic and phenolic groups) of the HA, which is 0.135 mM, therefore sufficient to completely neutralize the HA. Given the affinity, especially of the carboxylic groups to Ca^2+^, it is not surprising that here a rather strong neutralization by Ca^2+^ takes place that shifts the phase boundary substantially. From a practical point of view, this is important, as it reduces the amount of PDADMAC needed within the water treatment process, and it might be noted that a similar effect of reduced need for Al_2_(SO_4_)_3_ in the presence of Ca^2+^ has been reported before.^37^ Very interesting in this context is that the upper-phase boundary is not affected at all by the presence of Ca^2+^, which indicates that for polyelectrolyte excess, the phase behavior is dominated by the interaction with the polycation and Ca^2+^ is liberated from any binding site on the HA. For the overall phase behavior, it means that the width of the precipitation range substantially increases upon Ca^2+^ addition.

Interestingly, not only do the lower-phase boundaries shift but also the consistency of the precipitate changes. Compared to the reference system without Ca^2+^, the precipitates become more densely packed with enhanced structural stability that is less likely to be redispersed during agitation and transportation, as it might occur during industrial water treatment. These changes are evident in the confocal microscopy images shown in Figure S3 and inset in Figure 1, which show much larger and more compact precipitated flocs in the presence of Ca^2+^, typically forming agglomerates in the size range of 10–40 μm. In contrast, the flocs obtained in the absence of Ca^2+^ look much fluffier and less compact. In addition, the procedures of fine floc formation and agglomeration are observed much earlier during the mixing of HA and PDADMAC in the presence of Ca^2+^, i.e., Ca^2+^ speeds up the precipitation of humic acid. These changes indicate the capability of Ca^2+^ to lead to more effective precipitation.

### ζ-Potential

**Figure 2:**
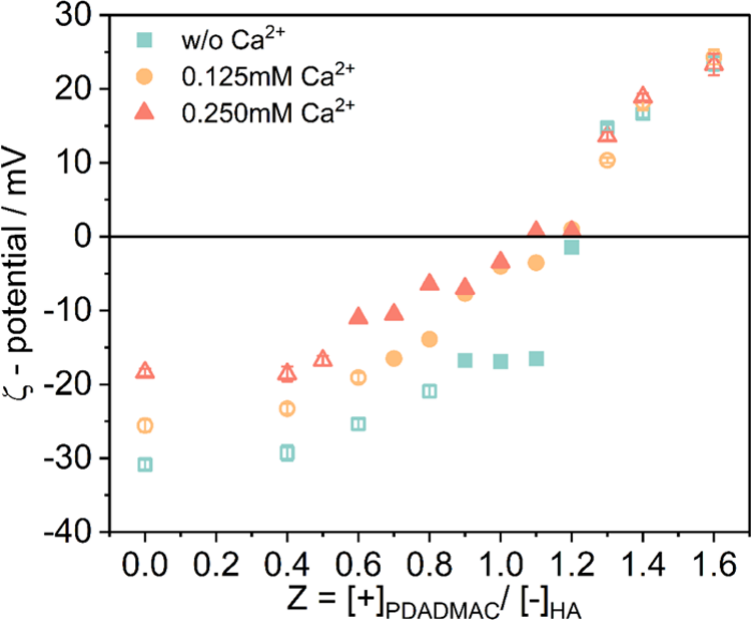
ζ-Potential for complexes of HA and 500 kDa PDADMAC for different Ca^2+^ concentrations at different charge ratios of *Z* (measurements done at pH 9 and 25 °C). Open symbols refer to monophasic regions, while full symbols refer to the biphasic region. Error bars are given or are smaller than the symbol size.

After having determined the macroscopic phase boundaries, we were now interested in quantifying the properties of the dispersed complexes as a function of the composition of the systems. For this, we measured the ζ-potential, which is the parameter quantifying the electrostatic repulsion between the particles caused by their ionized groups. It has been used for a long time in water treatment facilities to determine colloidal stability and to optimize coagulant dosages.^49,50^ This was done both in the monophasic regions and for the supernatant in the biphasic region, the latter being of key importance in the removal process during water treatment.^49,50^ The measured ζ-potential of all HA-PDADMAC complexes is shown in Figure 2, and tabulated values are given in Table S1 in the Supporting Information. The ζ-potential of pure HA solutions without added PDADMAC was negative, as expected, considering its functional groups (–COOH, phenolic, etc.). A continuous increase in the ζ-potential can be observed as cationic PDADMAC is added to the system. When the charge ratio *Z* reaches 0.9, the ζ-potential increases above −20 mV, i.e., into the range where interparticle repulsive forces become too weak to suppress coalescence or flocculation. With further addition of PDADMAC, the ζ-potential keeps increasing to over +10 mV and the system becomes monophasic again for Z above 1.3 due to the charge reversal taking place.

For HA solutions with added CaCl_2_, generally higher values of the ζ-potential are measured, becoming systematically higher with an increasing Ca^2+^ concentration. Apparently, the cationic Ca^2+^ is capable of complexing with HA molecules and neutralizing its charge to a certain degree. This overall increase of the ζ-potential is observed in the *Z* range up to 1.2, i.e., up to the upper boundary of the biphasic region. Interestingly, beyond this phase boundary, the presence of Ca^2+^ has no longer an effect on the observed ζ-potential. In the region of excess polycationic charge, the ζ-potential depends only on the dosage of PDADMAC and is independent of the concentration of Ca^2+^, which indicates that here Ca^2+^ is no longer bound to the aggregates and is substituted by the PDADMAC. Related specific prompting effects of Ca^2+^ under inadequate coagulant conditions only were also observed for HA removal with inorganic coagulants, such as aluminum sulfate (Al_2_(SO4)_3_) and modified polyaluminum chloride (PAC).^39^

### Removal Efficiency of Humic Acid

For the purpose of quantifying the concentration of humic acid in the supernatant of biphasic solutions with precipitate, we employed UV–vis spectrometry, as humic acid (HA) is known to absorb strongly in the UV range (see Figure S4 for a calibration curve). The absorbance at 254 nm (UV254) is a water quality test parameter that provides a quick measurement of the organic matter in water^51^ (of course, limited to humic substances and similar compounds with conjugated aromatic rings that absorb in that region). It is typically expressed as [*A*/*L*], where *A* refers to the measured value of UV254 and *L* is the optical path length, which is the thickness of the quartz cuvette. After having calibrated our system (for details, see Figure S4 in the Supporting Information), a linear correlation between HA concentration and UV254 was established, and the amount of HA in the supernatant of the biphasic region could be determined reliably, as given in Table S2 in the Supporting Information.

**Figure 3:**
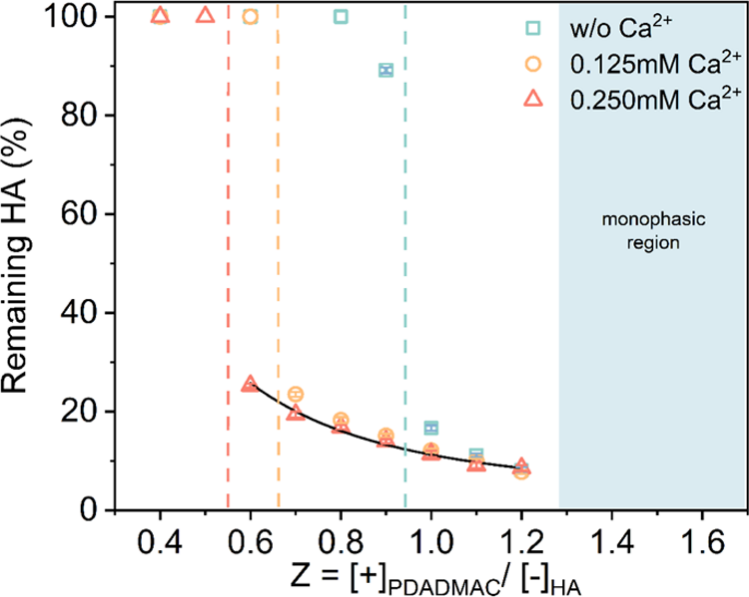
Remaining percentage of HA in the supernatant 24 h after mixing at different charge ratios *Z* at 25 °C at pH 9 in the presence of different concentrations of Ca^2+^ (open symbols: monophasic (100%), filled symbols: biphasic, crossed symbol: in a metastable state, partly precipitated). Error bars are given but are always smaller than the symbol size.

In Figure S5, the UV254 values of HA-PDADMAC systems 24 h after mixing are shown as a function of charge ratio *Z* for varying Ca^2+^ concentrations (precipitates settled under gravity). As the charge ratio *Z* increased from 0 to 1.6, a huge drop of the UV absorbance at 254 nm from ∼1.8 to below 0.4 was observed near the charge neutralization point (*Z* = 1), corresponding to a HA removal efficiency over 90%, showing that most of the humic acid was precipitated, and which compares well to previous observations for the similar process with much a higher *M*_w_ polycation.^26^ For the biphasic region, the concentration of the remaining HA was calculated and is shown in Figure 3. One observes that the concentration of remaining HA decreases continuously with increasing *Z*. For all systems with various Ca^2+^ concentrations, the highest removal efficiency was obtained next to the upper-phase boundary at *Z* = 1.2, corresponding to a remaining HA concentration of around 8% (here, the ζ-potential is close to 0). In agreement with the phase diagram (Figure 1), at lower *Z* values, higher HA removal can be caused by increasing the concentration of Ca^2+^. One also observes slightly lower values at a given *Z* with increasing Ca^2+^ concentration, but here, the Ca^2+^ effect is rather small. It is interesting to note that the HA removal efficiency as a function of the charge ratio *Z* keeps increasing until the monophasic region is reached again and without showing a minimum around the “equimolar charge point”.

This behavior can be described with the classical solubility product according to *K* = [HA] × [PDADMAC] (here, one may choose concentrations in mass per volume, as that is the concentration easiest known and which is directly proportional to the concentration of charged units). Assuming a simple precipitation behavior in which a certain percentage *x* of the PDADMAC relative to the humic acid (HA) becomes precipitated out of solution, one arrives at eq 11 (in the SI, we also give the equation rewritten in terms of *Z*), where the subscript 0 indicates the respective initial concentrations of PDADMAC and HA. Experimentally, we find from fitting this equation to the experimental data *K* equal to 57.4 mg^2^/L^2^ and a value of *x* of 0.249, which means as the charge ratio a value of 0.585, which is quite a bit below the potentially expected equimolar ratio and indicates that for the destabilization of the HA, only this larger fraction of PDADMAC is needed and the precipitate is in equilibrium with some excess PDADMA in solution. This model is in good agreement with the experiment data, as shown in Figure 3 as a solid line that describes very well the observed behavior within the precipitation range. Of course, the model is restricted to the biphasic regime, as for *Z* values lower or higher than the phase boundary, the complexes are colloidally stable again due to the excess PDADMAC. It might also be noted that *x* and *K* in eq 11 are to some extent interrelated with respect to their effect. Considering this and the finite precision of the [HA] values, we could convince ourselves that the error of the fitted values of *K* and *x* should be about 10%.
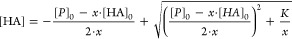
11

### Static and Dynamic Light Scattering (SLS, DLS)

In order to investigate the mesoscopic structure of HA-PDADMAC complexes, SLS and DLS measurements were carried out for HA-PDADMAC mixtures at a constant HA concentration of 40 mg/L in the *Z* range where samples remain monophasic for at least 24 h after mixing in the presence of different concentration of Ca^2+^.

**Figure 4:**
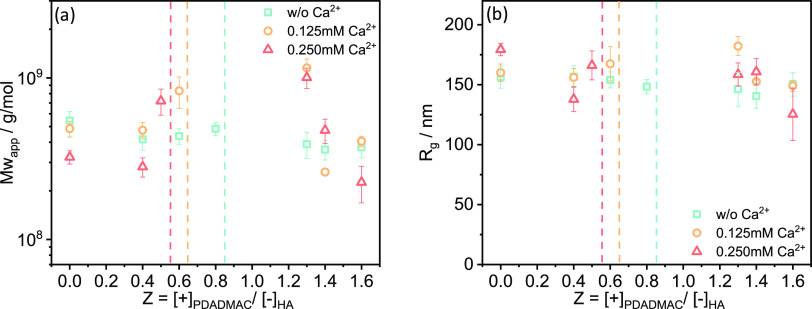
Static light scattering results: (a) Molecular weight *M*_w_ and (b) radius of gyration *R*_g_ for HA-PDADMAC complexes under varying Ca^2+^ concentrations as a function of the charge ratio *Z* at 25 °C.

The static intensity at zero angle, *I*_0_, was determined from a Guinier fit, and from it, molecular weight *M*_w_ and the radius of gyration *R*_g_ were calculated (see Figure S6 in the Supporting Information for SLS data). A slight increase in *I*_0_ of HA-PDADMAC complexes without Ca^2+^ can be observed as *Z* increases from 0 to 0.6, but the *M*_w_ of the aggregates remains almost constant, as shown in Figure 4a. This increase becomes more pronounced with a higher Ca^2+^ content, whereas the *M*_w_ for the samples of the pure HA (*Z* = 0) decreases with an increasing Ca^2+^ content. It is also interesting to note that the *M*_w_ for samples with Ca^2+^ is the highest for *Z* = 1.3, i.e., for the samples just beyond the biphasic region. Also, it is interesting to note that the *M*_w_ of the complexes does not vary very largely and remains in the range of 3–12 × 10^8^ g/mol. This value indicates that about 150–500 PDADMAC molecules must be contained in the complexes close to the phase boundary of precipitation.

The radii of gyration *R*_g_ of the formed complexes obtained from SLS (Figure 4b) show generally rather constant values of 140–170 nm, with a slight tendency to decrease with increasing charge ratio *Z*. Moreover, at *Z* = 0, where only HA molecules and Ca^2+^ are dispersed in solution, HA complexes with 0.25 mM Ca^2+^ show the biggest *R*_g_ values, which drop somewhat upon the addition of PDADMAC; whereas, the *R*_g_ of HA complexes without Ca^2+^ shows only minor variations with charge ratio *Z* changing from 0 to 0.4. Interestingly, it is observed that for the highest Ca^2+^ concentration of 0.25 mM, one sees for the pure HA solution a lower scattering intensity and a minimum at around (4–5) × 10^–2^ nm^–1^ (Figure S5c). This would indicate a less compact structure (likely globular) with about a 100 nm radius.

**Figure 5:**
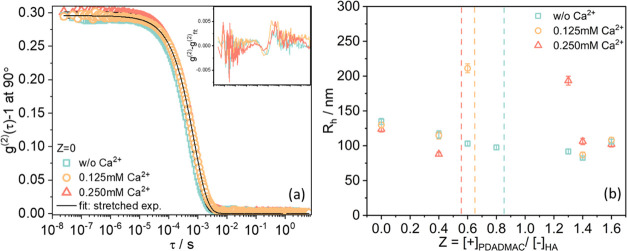
(a) Intensity autocorrelation functions from DLS experiments at 90° for HA solutions for varying Ca^2+^ concentrations at 25 °C and fits with a stretched exponential function (solid black line). The insets show the normalized fit residuals, (*g*^(2)^ – *g*_fit_^(2)^). (b) Apparent hydrodynamic radii *R*_h_ for HA-PDADMAC complexes under varying Ca^2+^ concentrations at different charge ratios *Z* at 25 °C. Error bars for *R*_h_ are given.

The DLS data generally show monomodal intensity correlation functions (Figure 5a for *Z* = 0 and Figures S8 for other *Z* values) that could be described well with a stretched exponential function (see eq 3). Values of the characteristic stretching exponent α for HA-PDADMAC complexes under varying Ca^2+^ concentrations at different charge ratios *Z* range between 0.80 and 0.95 and are summarized in Table S3, which indicates the presence of not too polydisperse aggregates. A pronounced decrease in α is observed for HA-PDADMAC complexes with 0.25 mM Ca^2+^ upon approaching the charge neutralization point. This means that the aggregates become more polydisperse in the vicinity of the phase boundary.

For pure HA, this analysis yields a rather constant value of the hydrodynamic radius *R*_h_ of ∼130 nm, irrespective of the Ca^2+^ concentration (Figure 5b). With the addition of PDADMAC, *R*_h_ of HA complexes decreases somewhat, which indicates a tendency for compaction, as *M*_w_ at the same time remains constant or even increases (Figure 4a). With further addition of PDADMAC, i.e., in the region of an excess polyelectrolyte, the aggregates are generally smaller than those of the pure HA and end up with similar size at *Z* = 1.6 (see Figure S8b in the Supporting Information), irrespective of the Ca^2+^ concentration. Apparently, here, PDADMAC takes the main role in complexing with HA, and, therefore, *R*_h_ is independent of the concentration of Ca^2+^, as previously seen from the constant upper-phase boundary and the values of the ζ-potential. Comparing the values of *R*_h_ (Figure 5b) and *R*_g_ (Figure 4b), we may conclude that the aggregates are rather open with respect to their structure, as for such more star-like shaped or highly branched aggregates, a higher value of *R*_g_ than for *R*_h_ is expected.^52^ The ratio of *R*_g_ and *R*_h_ is reported in Figure S9, with values of 1.2–1.6 found for all mixtures, except those near the phase boundaries. However, these are also a bit less reliable as samples might not be long-term stable, and typically precipitation is observed after several days. These rather high values for *R*_g_/*R*_h_ indicate the presence of more open and fluffy structures. It can also be noted that, in general, the value for *R*_g_/*R*_h_ increases with increasing *Z* value, thereby indicating that here, less homogeneous aggregates are formed.

In summary, SLS and DLS show rather constant radii in the range of 120–150 nm for the aggregates present, irrespective of the *Z* value and the addition of Ca^2+^.

Besides these DLS measurements for samples that remain monophasic for at least 24 h, the HA-PDADMAC mixtures in the biphasic region, at *Z* = 1.0 for varying Ca^2+^ concentrations, were also measured 5 min after mixing, in order to look into the development of complexation at the initial stage (intensity autocorrelation functions are shown in Figure S10). Here, the reliability of the data is not so high since precipitation sets in rapidly, and sedimentation may affect the DLS results. However, the intensity correlation functions clearly show much longer correlation times for Ca^2+^ addition, even after 5 min of mixing, indicating a fast formation of larger agglomerates. The hydrodynamic radii are 345 nm without Ca^2+^ and 3630 and 5215 nm for 0.125 and 0.25 mM Ca^2+^, respectively, i.e., becoming much larger with increasing Ca^2+^ concentration. This difference initiated our interest in the effect of Ca^2+^ on the rate of coagulation and floc formation, as well as the rate of settling of flocs, which will be discussed later in the manuscript.

### Small-Angle Neutron Scattering (SANS)

When light is adopted as the probing radiation, the q-range is limited by the wavelength of the incident light, with which only the larger scale of HA-PDADMAC complexes can be investigated. As neutrons have a much longer wavelength compared to lights, SANS experiments were performed to further determine the mesoscopic structure of the soluble HA-PDADMAC complexes within the monophasic region with higher local resolution. The results from SLS represent the large-scale size of the aggregate, while the results from SANS indicate the internal arrangement of local structures within a given aggregate. Figure S11 displays the scattering pattern of HA-PDADMAC complexes obtained by plotting together SLS (low-*q* data) and SANS measurements (SANS curves are in absolute units, and the SLS data were normalized to the SANS data by taking into account the corresponding contrast factor; for details, see SI). There is good agreement of the absolute values and relative tendencies for both SANS and SLS curves, and this combined plot shows the finite size of the aggregates studied by approaching a plateau in SLS, while SANS just sees a smaller size scale on which the matter is distributed according to a fractal law. Basically, SLS and SANS were used as complementary techniques to offer a more comprehensive understanding of the overall structure of HA-PDADMAC complexes.

It should be noted that in order to achieve adequate scattering intensity, a much higher concentration of 1.0 g/L HA was utilized in the SANS experiments. This increase in the concentration of the system also leads to a broader biphasic region (see Figure S12), which now ranges from *Z* = 0.8 to 1.4 without Ca^2+^ and *Z* = 0.2 to 1.4 for 3.125 mM Ca^2+^ (same HA/Ca ratio as for the 40 mg/L HA system with 0.125 mM Ca^2+^). Accordingly, the charge ratio of samples selected for SANS differs slightly compared to that of the series for LS described above. For a broader comparison of the cation effect, also samples with identical ionic strength with NaCl or MgCl_2_ as salts were prepared. Figure S12 shows the visual appearance of the measured samples, and one observes the much faster precipitation in the case of addition of Ca^2+^ and partly for Mg^2+^.

**Figure 6:**
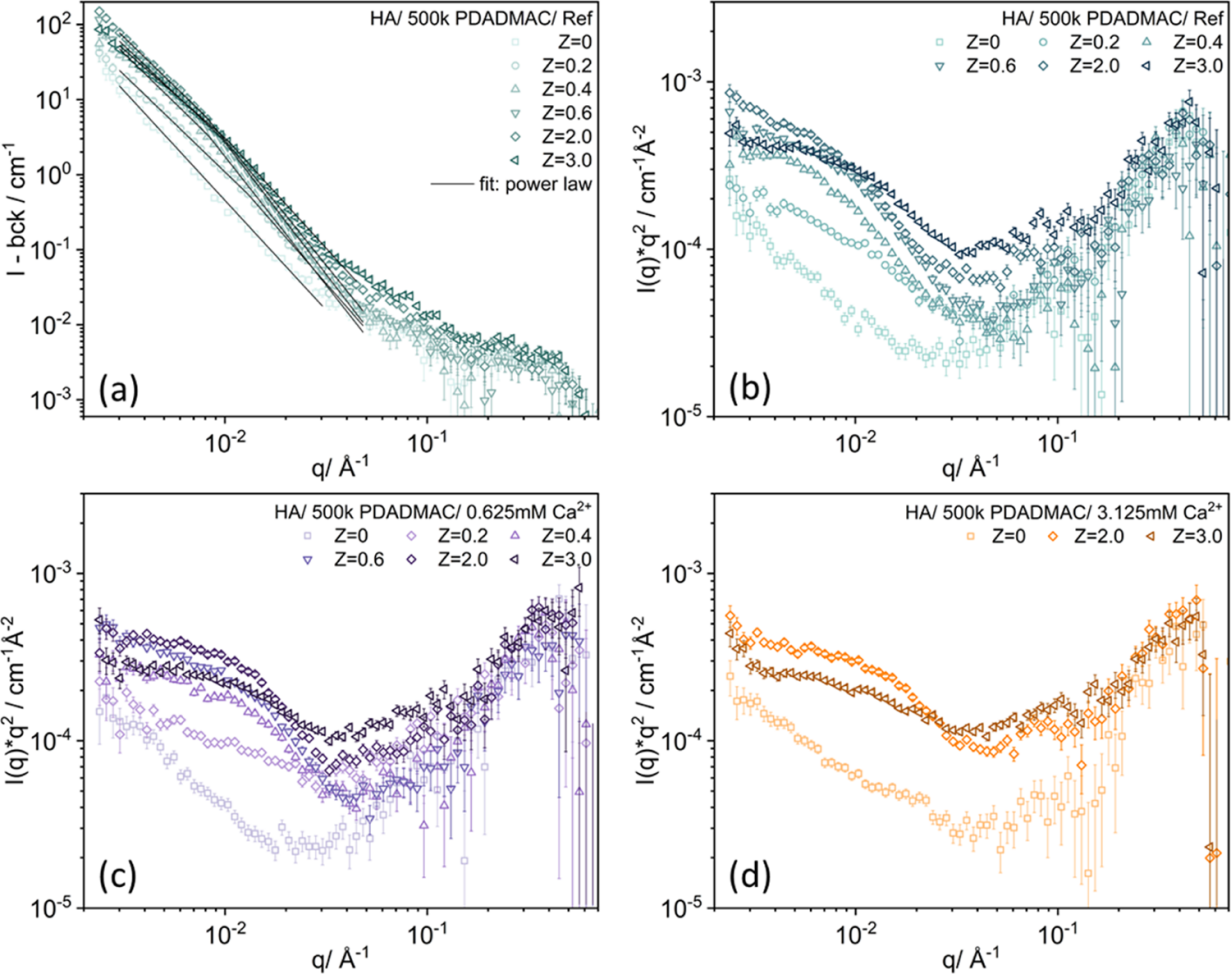
SANS intensity as a function of the magnitude of the scattering vector *q* for complexes of HA and 500 kDa PDADMAC (a) without added salt (the curves with added CaCl_2_ are shown in Figure S13). Kratky–Porod plots for (b) without added salt, (c) with 0.625 mM CaCl_2_, and (d) with 3.125 mM CaCl_2_ (all measured at 25 °C).

Figures 6 and S13 show the SANS data of complexes formed by 1.0 g/L HA and different amounts of 500 kDa PDADMAC with mixing ratios *Z* ranging from 0 to 3 in the presence of different concentrations of CaCl_2_ and without it. For the pure HA-PDADMAC system with no added salt, an increase in scattering intensity at lower *q* is observed with increasing charge ratio from 0 to 2, followed by a decrease in scattering intensity as *Z* reaches 3 (Figure 6a). That can be attributed to the formation of bigger complexes, and in addition, the total polymeric concentration increases due to the addition of the PDADMAC. The scattering curves at lower *q* also show a transition from the *q*^–3^ power law behavior characteristic for a pure humic acid solution to *q*^–2.6^ behavior with increasing *Z* before reaching the phase boundary. Beyond the phase boundary, this trend continues further to *q*^–2.3^, indicating the formation of apparently less densely packed structures on this larger size scale with increasing PDADMAC addition. In contrast, the slope at higher q increases with increasing *Z* until the phase boundary is reached and then for *Z* values beyond the phase boundary decreases again. This means that the complexes become more compact on a local scale in the vicinity of the biphasic region.

The intensity and slope changes are seen more clearly in the Kratky–Porod plot shown in Figure 6b–d. The presence of 0.625 mM Ca^2+^ has only a small effect on the phase behavior, and also the scattering curves (Figure 6c) look very similar to the case without added Ca^2+^ (Figure 6b). In contrast, for addition of 3.125 mM CaCl_2_, precipitation at *Z* = 0.2, 0.4, and 0.6 happens quickly; thus, only complexes in the PDADMAC excess region and pure HA–Ca^2+^ complexes were measured. However, the SANS curves at *Z* = 2.0 and 3.0 look quite similar (Figure 6d), which means that the addition of the Ca^2+^ has only a little effect on the complex structure.

More details of the effect of PDADMAC addition are seen upon dividing the scattering data for different *Z* values by the data obtained for *Z* = 0 (Figure S14a–c). For the case of no added Ca^2+^ and 0.625 mM Ca^2+^, one clearly sees a marked increase of scattering intensity in the mid-*q*-range (0.005–0.02 Å^–1^) that becomes more marked with increasing PDADMAC addition and then is most pronounced for samples beyond the biphasic region, i.e., for *Z* = 2.0 and 3.0, where the scattering intensity increases by about a factor 8. This indicates that upon PDADMAC addition, much more compact structural features in the size range of 10–50 nm are formed. In contrast, for the highest Ca^2+^ content (3.125 mM CaCl_2_), a much less pronounced increase in scattering intensity is seen (Figure S14c). The effect of Ca^2+^ addition on the structure of the pure HA aggregates (*Z* = 0) is seen more obviously in the Kratky–Porod plot, as shown in Figure S14d; in the *q*-range below 0.05 Å^–1^, the intensity is significantly higher for 3.125 mM Ca^2+^. This means that at higher Ca^2+^ content, the structures of the HA are already modified in a certain way that then is not much affected by PDADMAC addition, and mostly, the PDADMAC is attached to the existing structures.

For the case of *Z* = 2.0 and 3.0, one can learn more about the effect of Ca^2+^ by comparing directly to the curves without added CaCl_2_ (Figure S15). For *Z* = 2.0, the presence of Ca^2+^ leads to a peak at ∼0.02 Å^–1^ that becomes somewhat more prominent for higher CaCl_2_ concentration, while at lower *q*, the addition of Ca^2+^ leads to a reduction in scattering intensity. For *Z* = 3.0, a similar behavior is seen but much less pronounced, and the peak is shifted to a higher *q* of ∼0.035 Å^–1^. This can be interpreted such that here, the addition of Ca^2+^ leads to a local ordering of the aggregated mass on a scale of ∼30 nm.

To elucidate in more detail generic vs specific ion effects on the mesoscopic structure of the complexes, in further experiments, CaCl_2_ was replaced by NaCl and MgCl_2_ while retaining the ionic strength at 9.375 mM. Consistent with the previous literature claiming that Ca^2+^ has a much larger effect than Mg^2+^ on HA aggregation, macroscopic precipitation was observed in a much narrower range of charge ratios *Z* when replacing Ca^2+^ with Mg^2+^, as shown in Figure S12. This indicates much weaker binding of Mg^2+^ to humic acid as reported previously.^34^ When replaced by Na^+^, precipitation was further suppressed, as no or much weaker intra- or intermolecular bridges should be formed with monovalent ions.

**Figure 7:**
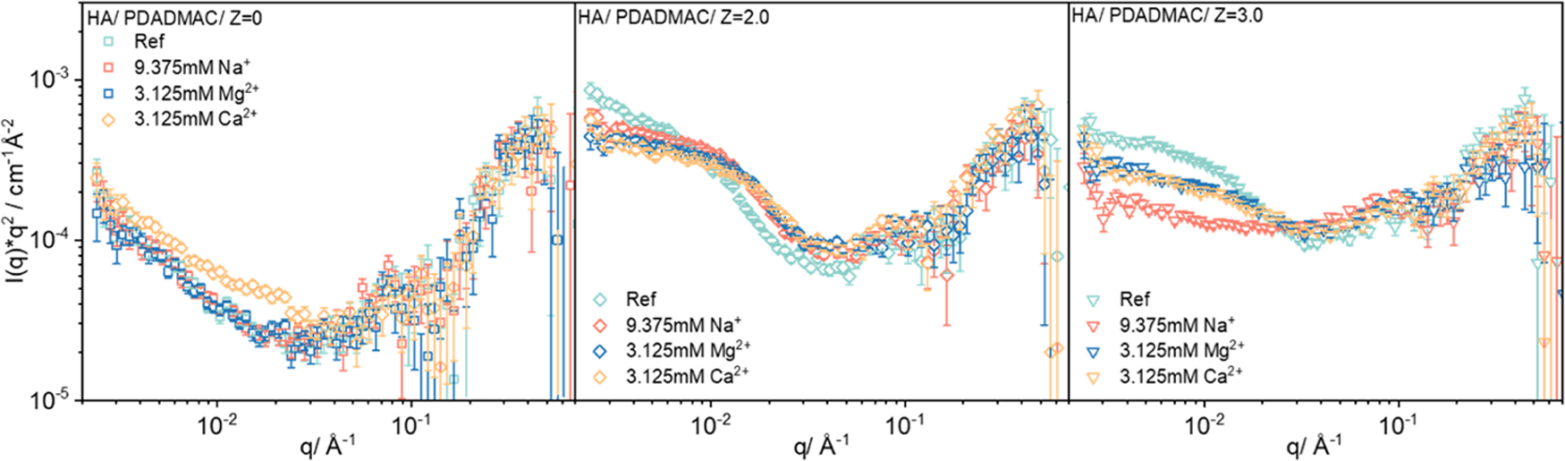
SANS intensity *I* × *q*^2^ as a function of the magnitude of the scattering vector *q* for complexes of HA and 500 kDa PDADMAC under various ionic conditions with *Z* = 0, 2, and 3, respectively.

The scattering curves for the samples with different *Z* are summarized in Figure S16. One observes rather similar changes as a function of charge ratio *Z* for the different HA-PDADMAC complexes at all ionic conditions, irrespective of whether using NaCl, MgCl_2_, or CaCl_2_ (Figure 6). When grouping together the results at the same *Z* values (see Figures 7 and S17 in the Supporting Information) for the different salts, the differences can be seen more clearly. For the simple system of pure HA (ref) and different salts, no obvious change in SANS can be seen compared to pure HA solutions; only a somewhat higher scattering intensity is observed for Ca^2+^. This becomes better visible when normalized by the intensity of pure HA without added salt (*I*/*I*_ref_), as shown in Figure S18. This suggests the formation of somewhat more compact complexes here due to the presence of Ca^2+^. Furthermore, as *Z* increases from 2 to 3, a much more drastic intensity drop at lower q is seen for HA-PDADMAC-Na^+^ complexes (Figure 7), which indicates that for the formation of larger complexes, bivalent cations are required. The observed ion specificity aligns generically with the well-established Hofmeister effect.^53^ Influenced by the strength and interaction of hydration shells, distinct aggregation behavior of HA in the presence of Na^+^, Mg^2+^, and Ca^2+^, respectively, can be observed. However, the distinct role played here by Ca^2+^ certainly arises from its strong interaction with the carboxylic groups of the HA. Certainly, also a further theoretical analysis of the electrostatic conditions in such complexes and during their formation process would be interesting but is largely hampered by the rather ill-defined mesoscopic structure of humic acid and, therefore, appears out of scope at the given moment.

For a more quantitative interpretation, the SANS data of the HA-PDADMAC complexes was analyzed empirically by a two-power law model (eq 5) with all obtained parameters summarized in Table 1. For *Z* = 0, i.e., a pure humic acid solution, under various ionic conditions, only a single power law is observed, where the exponent *p* decreases only somewhat by switching from Na^+^, Mg^2+^ to Ca^2+^, where the most strongly binding Ca^2+^ shows the lowest value. As soon as PDADMAC is added, a switch of the slope is seen in the *q*-range of 0.01–0.1 Å^–1^, the curve becoming steeper at higher *q*. Without added salt, the initial slope (*p*1) is rather unaffected by the addition of PDADMAC and only becomes significantly smaller for *Z* = 3.0. In contrast, the second slope (*p*2) increases substantially upon PDADMAC addition until the phase boundary is reached and for the *Z* values beyond becomes smaller again. In the presence of Na^+^, the same tendencies are seen, just with generally somewhat lower values. The addition of Mg^2+^ has only a very small effect on *p*1, but markedly higher values for *p*2 are seen for *Z* values below the phase boundary, where the higher fractal dimension indicates a more compact structure in that size range. For Ca^2+^, only the *Z* range beyond the phase boundary was studied and shows here very similar values as seen for Mg^2+^. Interesting is the observation that for complexes at *Z* = 3, the addition of salt always leads to a substantial reduction of the slope at higher q (Table 1), which is most pronounced for the case of Na^+^. In general, this means that salt here leads to less compact aggregates on the length scale of 100–200 nm.

**Table 1:** Fitted Power Law Exponent *p*1 at Low *q* and *p*2 at High *q*, Respectively, of HA Complexes with Different Charge Ratios *Z* Values under Different Ionic Conditions, as Determined from the SANS Experiments

	0	0.2		0.4		0.6		2		3	
Z	*p*	*p*1	*p*2	*p*1	*p*2	*p*1	*p*2	*p*1	*p*2	*p*1	*p*2
ref	2.92	2.59	3.08	2.63	3.43	2.55	3.51	2.61	3.33	2.35	2.95
Na	2.9	2.44	2.98	2.44	3.30	2.52	3.47	2.31	3.25	2.29	2.08
Mg	2.86	2.57	3.27	2.60	3.81			2.30	3.12	2.32	2.48
Ca	2.76							2.25	2.98	2.29	2.50

As an alternative approach, we analyzed our data with the Beaucage model to interpret the hierarchical structural levels of HA complexes, and the corresponding fit parameters are summarized in Table S4. Corresponding residuals for the fit of SANS data for 500 kDa PDADMAC-HA complexes under various ionic conditions at *Z* = 2 with the two-power law model and the Beaucage model, respectively, are shown in Figures S19 and S20, as an example. Of course, in general, the Beaucage is superior with respect to its fit quality; especially for a pure humic acid solution under various ionic conditions, a one-level Beaucage model was adopted, where the radius of gyration *R*_g1_ shows comparable values at around 44 nm for the pure HA as a reference system. The addition of Na^+^ or Mg^2+^ ions has no effect on this size, while the more strongly binding Ca^2+^ leads to an increase of *R*_g1_ to 56.1 nm, indicating a “bridging effect” between HA molecules and Ca^2+^ even in the absence of a cationic electrolyte.

As discussed in previous sections, the addition of PDADMAC leads to a local densification of the structures in the complexes, and the two-level Beaucage model was utilized for the sample sets where PDADMAC was involved (Table S4). For the pure HA-PDADMAC system with no added salt, a steady increase for both *R*_g1_ and *R*_g2_ is observed as the charge ratio increases from 0 to 3, confirming the formation of bigger complexes due to the addition of PDADMAC. Here, a jump-wise increase from 44 to 56 nm occurs already upon the addition of the smallest amount of PDADMAC, and then *R*_g1_ increases systematically up to 95.5 nm for the sample with PDADMAC excess. At the same time, *R*_g2_ starts from 5.2 and goes up to 9.6 nm, being almost constantly a factor 10 smaller than *R*_g1_. The appearance of this smaller scale structure indicates that the complexation by PDADMAC also leads to a densification of structures on this rather local level of 5–10 nm, which becomes larger with increasing amount of complexing PDADMAC. This is further corroborated by the fact that G_2_, which quantifies the presence of these smaller compacted structures, increases substantially with increasing *Z* (Table S4).

With the addition of Na^+^, the HA-PDADMAC complexes *R*_g1_ and *R*_g2_ follow a similar tendency until charge ratio 2, i.e., until the phase boundary is reached and above one no longer observes the increase of *R*_g1_. When switching from the monovalent Na^+^ to bivalent Mg^2+^, a pronounced higher value for *R*_g1_ of 108 nm is seen when *Z* approaches the phase boundary, followed by a rapid drop of the *R*_g1_ value by a factor of around 2 at the PDADMAC excess region, while the radius of gyration at smaller scale *R*_g2_ is less influenced by the charge ratio *Z*. For HA-PDADMAC complexes at both ionic conditions, at a higher *Z* value, where PDADMAC is in excess, the considerable reduction of *R*_g1_ compared to the salt-free condition indicates the shrinkage of HA complexes in the length scale of 50–100 nm, while the comparable *R*_g2_ suggests that the structural rearrangement on a smaller scale is little affected. For Ca^2+^, due to precipitation, no soluble complexes could be studied with PDADMAC, but for HA-PDADMAC excess, interestingly, the values for *R*_g1_ and *R*_g2_ are even a bit bigger than those without added Ca^2+^. As an explanation, one could state that apparently, for PDADMAC excess, the ionic strength brought into the system by addition of Na^+^ or Mg^2+^ weakens the binding of PDADMAC and HA, while for Ca^2+^, the effect of ionic strength increase is counterbalanced by its ability to contribute to bridging of HA molecules, thereby keeping the complexes large

### Settling Rate of the Humic Acid Precipitate

So far, we have been concerned with the equilibrium or steady-state behavior of the HA-PDADMAC complexes under various conditions. However, equally important for the process of HA removal by precipitation is the rate at which this takes place, i.e., the rate at which the coagulation takes place and the precipitate forms and settles.

**Figure 8:**
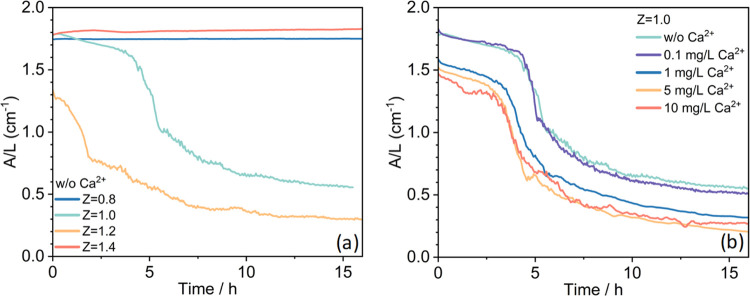
Time dependence of the HA-PDADMAC mixture absorbance at 254 nm with various (a) charge ratios *Z* (addition of PDADMAC) and (b) for different Ca^2+^ concentrations at fixed charge ratio *Z* = 1.

To monitor these processes, the time-dependent absorption changes at 254 nm of HA-PDADMAC mixtures were measured over a longer time period of 16 h, starting 3 min after mixing. The results are shown in Figure 8. For charge ratios *Z* = 0.8 and *Z* = 1.4, where no precipitation happened, constant absorbance values are observed throughout the whole 16 h observation window. The slightly higher absorbance at *Z* = 1.4 is likely due to the higher concentration of dispersed material. For *Z* = 1.0 and *Z* = 1.2, precipitation can be observed, and for these Z values, HA removal efficiencies at around 83 and 92%, respectively, were achieved within 24 h after mixing (Figure 3). For these samples, a gentle decline of absorbance at 254 nm can be observed at the very beginning, followed by a much more rapid decrease after 1.5 or 4 h, respectively. For longer times, the absorbance values decreased much more slowly and after 10–15 h, reached values of around 0.5 and 0.3, respectively, which correspond to removal efficiencies of about 70 and 80%, respectively (Figure 8a; the values here after 15 h are a bit lower than those after 24 h (Figure 3) as remaining dispersed flocs still settle during that time). At this moment, the major part of HA was already precipitated out and phase separation could be observed clearly, with a tiny amount of HA flocs remaining in the supernatant that will settle after longer times (see Figure 3). Interesting to note is that a slight excess of PDADMAC (*Z* = 1.2) makes the precipitation process proceed much faster and more efficiently than under apparent equimolar conditions (*Z* = 1.0).

We then investigated further the effect of Ca^2+^ on the precipitation process. In Figure 8b, we compare the absorbance (of the developing supernatant) as a function of time for the system at *Z* = 1.0. Generically, a similar decline-drop-plateau curve of the absorbance at 254 nm can be seen. However, already the addition of 1 mg/L Ca^2+^ (0.025 mM), which corresponds to around one Ca^2+^ ion per 5.5 carboxylic groups from the HA molecules, leads to a marked speeding up of the settling process. It is comparable to the one for a 20% higher dosage of PDADMAC (*Z* = 1.2, Figure 8a), indicating the Ca^2+^ effect in promoting HA precipitation. The observed calcium effect on HA precipitation, as measured through the time-resolved UV–vis technique, was systematically reproduced to ensure the reliability and consistency of our results. In summary, this study shows that the presence of Ca^2+^ during the precipitation process leads to a more marked removal of HA and also speeds up this removal process. Both effects are of high relevance to the practical use of polycations in the NOM removal in the water industry.

## Conclusions

In this work, we systematically studied the interaction between oppositely charged humic acid (HA) and polycation polydiallyldimethylammonium chloride (PDADMAC), which is widely used in conventional water treatment, with a particular focus on the effect of Ca^2+^ on the complexation process. The complexation of HA by polycations is an elementary step in water treatment, in which Ca^2+^ will always be present to a certain extent. All of these investigations were done at pH 9.0 to mimic the realistic conditions in a water plant. To obtain a comprehensive view of the behavior of this system, the ion concentration and the charge ratio *Z* between the HA and PDADMAC were varied.

A combination of UV–vis and confocal microscopy was utilized to probe and quantify the phase behavior and morphology of the formed HA–PE precipitates as a function of the mixing ratio for various Ca^2+^ concentrations. This investigation showed that the biphasic region is shifted to a lower charge ratio *Z* already for rather small Ca^2+^ concentrations of 0.125 mM (actually close to the 0.135 mM of charged HA units contained in our experiments), which means that a significantly lower amount of PDADMAC is needed for achieving the same effect. At the same time, the packing density of HA flocs in the precipitate was enhanced by the presence of Ca^2+^, which is presumably relevant to the floc strength of the precipitates. The amount of precipitated HA in the two-phase region is well described by a simple solubility law and does not depend to a significant extent on the presence of Ca^2+^. In addition to the phase behavior, for HA-PDADMAC complexes with a common charge ratio *Z* = 1, a significantly faster precipitation process in the presence of Ca^2+^ was quantitatively verified via long-term UV–vis measurements.

**Figure 9:**
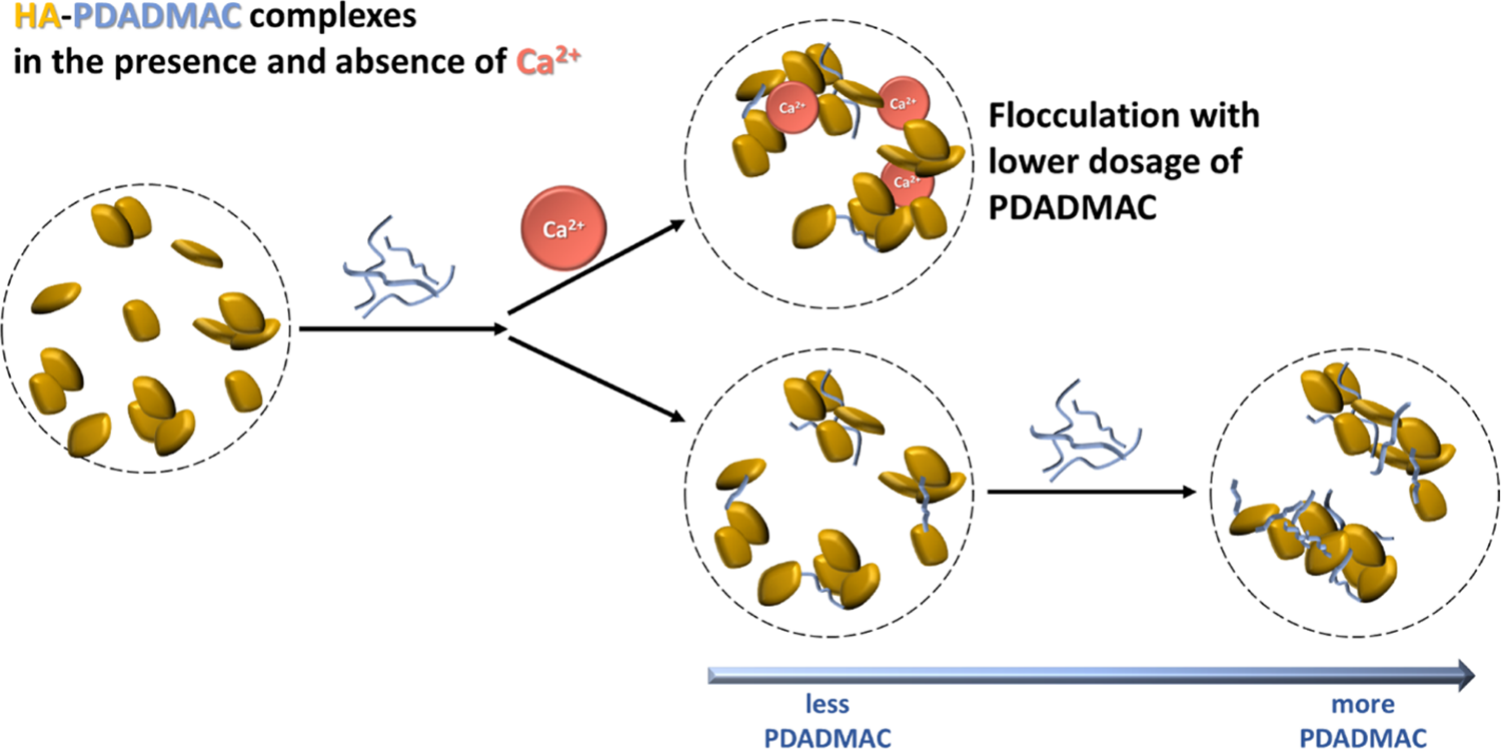
Schematic drawing of the complexation between cationic PDADMAC and anionic HA in the presence and the absence of Ca^2+^, respectively.

Moreover, the colloidal structural characteristics of HA-PDADMAC complexes, to the extent of our knowledge, were, for the first time, studied by comprehensive scattering measurements, including SLS, DLS, and small-angle neutron scattering (SANS). A slight decrease of the radii of gyration *R*_g_ of the formed complexes with increasing charge ratio *Z* was shown by SLS measurements, which became more pronounced in the presence of Ca^2+^. As the calculated *R*_g_/*R*_h_ values are largely above 0.775, it implies a rather open structure of the HA-PDADMAC complexes. Further, one has the “bridging effect” from the bivalent ion Ca^2+^, and Figure 9 depicts the complexation in the presence and absence of Ca^2+^. The SANS experiments showed a markedly more compacted structure in the size range of 10–50 nm due to the addition of PDADMAC. Interestingly, the addition of PDADMAC reduces the fractal dimension on a larger scale but increases it for smaller sizes, which demonstrates the local densification of the structures in the complexes. Here, the presence of Ca^2+^ has only a minor effect, but the SANS experiments show a clearly discernible ordering effect in the size range of 30 nm at PDADMAC excess. In summary, this means that the main structural effect of PDADMAC addition is a compaction of aggregates in the structural range of 20–50 nm, further supported by the presence of Ca^2+^. In order to elucidate the specific ion effects in more detail on the mesoscopic structure of the complexes, HA-PDADMAC complexes under more ion conditions were probed with SANS, where the difference between the effect of monovalent ions and bivalent ions was clearly revealed. These experiments also showed that Ca^2+^ plays a special role here due to its very marked interaction with the carboxylic groups of the HA.

These insights from colloid science may shed light on the optimization of the water treatment process in industrial fields. Also, it provides a reference characterization of HA–PE complexes as a basis for the development of PE from natural resources to develop an environmentally friendly solution.

## Supporting Information Available

Potentiometric titration curve of humic acid; photo of samples; confocal laser scanning microscopy (CLSM) images of precipitated flocs; detailed description of UV254; additional light scattering results; and additional SANS results (PDF)
