# Supplementary material for: Colloidal Chemistry in Water Treatment: The Effect of Ca2+ on the Interaction between Humic Acid and Poly(diallyldimethylammonium chloride) (PDADMAC)
Source: Langmuir. 2024 Feb 19;40(8):4108–21. doi: 10.1021/acs.langmuir.3c03029 (PMC10906001; doi:10.1021/acs.langmuir.3c03029)
Supplement: Supplementary file 1 — la3c03029_si_001.pdf [file la3c03029_si_001.pdf]

Supporting Information

Colloidal Chemistry in Water Treatment: The Effect of Ca^{2+} on the Interaction Between Humic Acid and poly (diallyldimethylammonium chloride) (PDADMAC)

Mingyu Yuan [1,], Heriberto Bustamante [2], Najet Mahmoudi [3], Michael Gradzielski[1,*]*

1: Stranski-Laboratorium für Physikalische und Theoretische Chemie, Institut für Chemie, Technische Universität Berlin, D-10623 Berlin, Germany

2: Sydney Water, Parramatta NSW 2125, Australia

3: ISIS Neutron and Muon Source, Rutherford Appleton Laboratory, Harwell Oxford, Didcot OX11 0QX, United Kingdom

*Corresponding author: michael.gradzielski@tu-berlin.de； m.yuan.1@campus.tu-berlin.de

Number of Pages: 20

Number of Figures:20

Table of Contents

1. Materials.. 3

2. Phase behaviour – Variation of Ca^{2+} concentration.. 4

3. Zeta-potential 5

4. Removal efficiency of humic acid – UV-vis monitoring.. 5

5. Static and Dynamic Light Scattering (SLS, DLS) 8

6. Small-Angle Neutron Scattering (SANS) 11

1. Materials

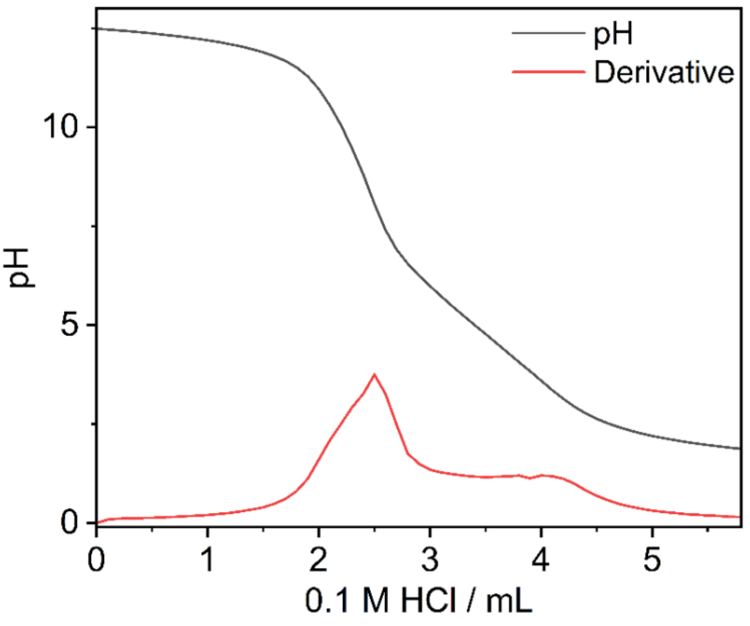

Figure S1. Potentiometric titration curve of humic acid with a solution of HCl.

2. Phase behaviour – Variation of Ca^{2+} concentration

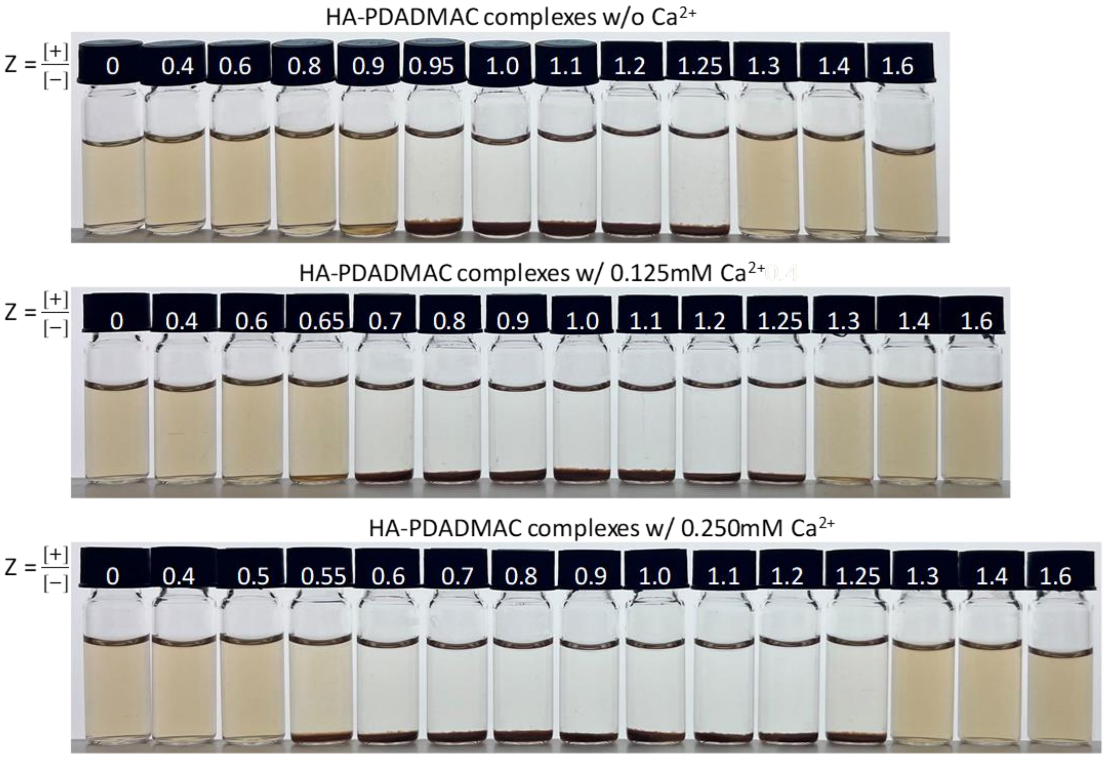

Figure S2. Photos of samples containing 40 mg/L HA at different charge ratio Z at pH 9 and for varying Ca^{2+} concentrations.

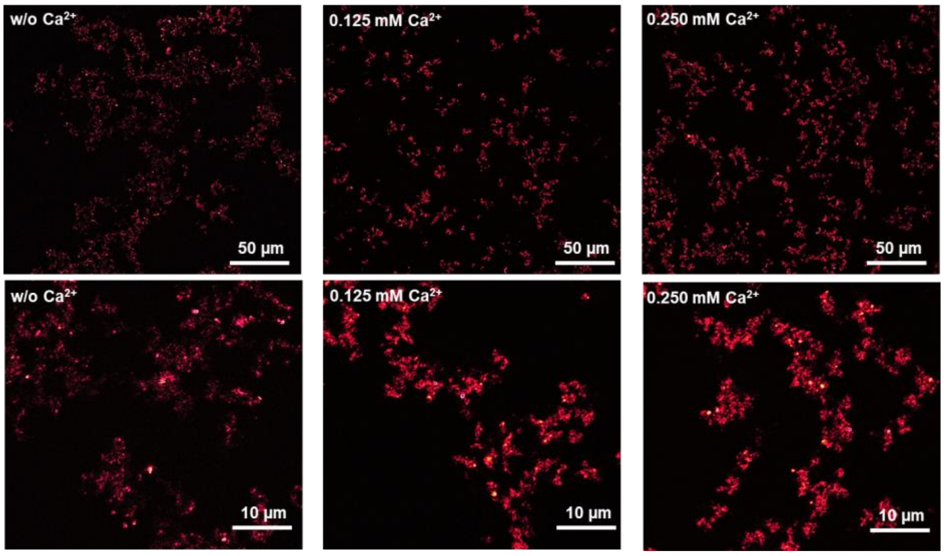

Figure S3. Confocal laser scanning microscopy (CLSM) images of precipitated flocs at Z = 1.0 for varying Ca^{2+} concentrations.

3. Zeta-potential

Table S1. ζ-potential for complexes of HA (40 mg/L) and 500 kDa PDADMAC for different Ca^{2+} concentrations at different charge ratio Z (T = 25 °C).

**Z**	**0**	**0.4**	**0.5**	**0.6**	**0.7**	**0.8**	**0.9**	**1**	**1.1**	**1.2**	**1.3**	**1.4**	**1.6**
	ζ, mV	ζ, mV	ζ, mV	ζ, mV	ζ, mV	ζ, mV	ζ, mV	ζ, mV	ζ, mV	ζ, mV	ζ, mV	ζ, mV	ζ, mV
w/o Ca^{2+}	-30.9	-29.3		-25.3		-20.9	-16.8	-16.9	-16.5	-1.4	14.7	16.6	23.4
	(2.4)	(1.1)		(0.7)		(0.8)	(0.6)	(1.1)	(0.6)	(0.5)	(0.4)	(0.5)	(0.9)
0.125mM Ca^{2+}	-25.6	-23.3		-19.1	-16.5	-13.9	-7.7	-4.0	-3.5	1.0	10.3	18.3	24.3
	(0.8)	(0.7)		(0.7)	(1.3)	(1.2)	(0.7)	(0.6)	(0.2)	(0.5)	(0.4)	(1.1)	(1.0)
0.250mM Ca^{2+}	-18.4	-18.6	-16.8	-11.0	-10.5	-6.5	-7.0	-3.5	0.7	0.7	13.6	18.9	23.3
	(0.5)	(1.0)	(0.6)	(0.6)	(0.8)	(0.7)	(0.2)	(0.4)	(0.4)	(0.5)	(0.6)	(0.5)	(1.5)

4. Removal efficiency of humic acid – UV-vis monitoring

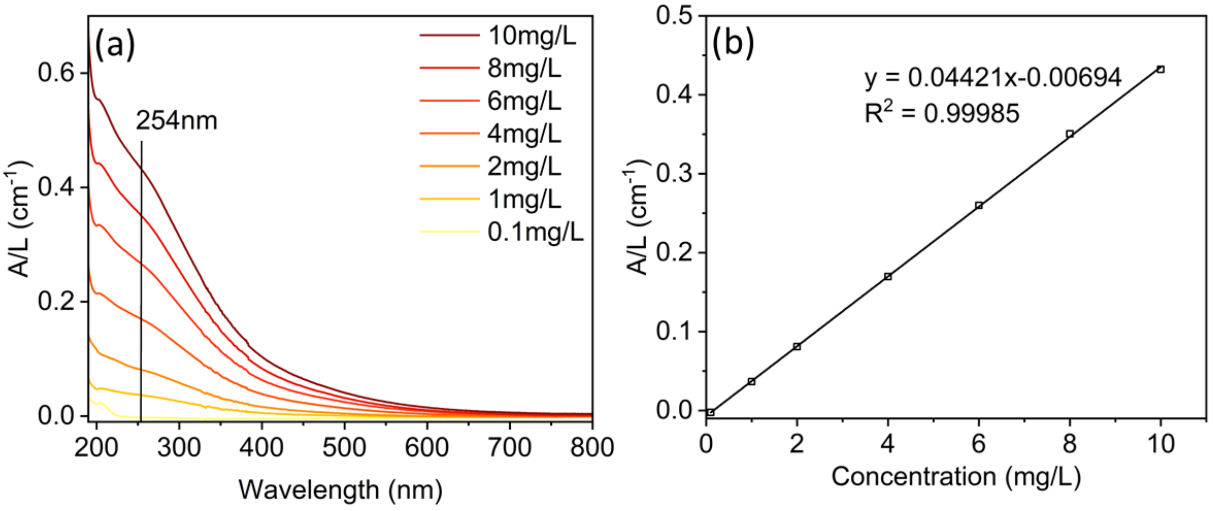

Figure S4. (a) UV-vis spectra of humic acid solutions of different concentrations at pH = 9. (b) Calibration curve for HA concentration as a function of UV absorbance at 254 nm.

Table S2. Decadic UV-absorbance at 254 nm per optical path length (UV254) of HA-PDADMAC systems.

Z	w/o Ca^{2+}			0.125 mM Ca^{2+}			0.250 mM Ca^{2+}		
	A/L	HA conc	Removal efficiency	A/L	HA conc	Removal efficiency	A/L	HA conc	Removal efficiency
	cm^{-1}	mg/L	%	cm^{-1}	mg/L	%	cm^{-1}	mg/L	%
**0.4**	1.78			1.76			1.77		
**0.5**							1.77		
**0.6**	1.79			1.79			0.44	10.1	74.7
**0.7**				0.41	9.4	76.5	0.34	7.8	80.6
**0.8**	1.81			0.32	7.3	81.7	0.29	6.7	83.2
**0.9**	1.57	35.7	10.8	0.26	6.0	84.9	0.24	5.6	86.0
**1**	0.29	6.7	83.4	0.21	4.8	87.9	0.19	4.6	88.6
**1.1**	0.19	4.4	88.9	0.17	4.0	90.0	0.15	3.6	90.9
**1.2**	0.14	3.2	91.9	0.13	3.1	92.3	0.14	3.4	91.5
**1.3**	1.73			1.68			1.83		
**1.4**	1.77			1.75			1.76		
**1.6**	1.78			1.79			1.80		

*the supernatant of samples was measured 24 hours after mixing for different Ca^{2+} concentrations at different charge ratio Z at 25 °C. For the biphasic region the corresponding HA concentration and removal efficiency were calculated.

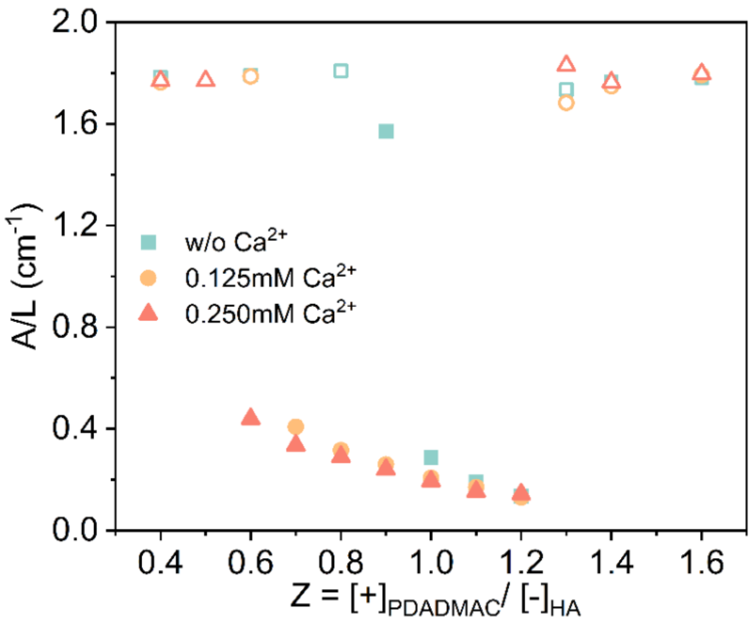

Figure S5. Decadic UV-absorbance at 254 nm per optical path length (UV254) of HA-PDADMAC systems (whole solution for monophasic region and supernatant for biphasic region) 24 hour after mixing under varying Ca^{2+} concentrations at different charge ratio Z at 25 °C. The HA concentration was 40 mg/L and the pH 9.0.

Phase behaviour described with the classical solubility product

According to classical solubility product K = [HA]*[PDADMAC] (concentrations in mass per volume), assuming a simple precipitation behaviour in which a certain percentage x of the PDADMAC relative to the humic acid (HA) becomes precipitated out of solution, one arrives

$$[HA] = -\frac{[P]_0 - x \cdot [HA]_0}{2 \cdot x} + \sqrt{\left(\frac{[P]_0 - x \cdot [HA]_0}{2 \cdot x}\right)^2 + \frac{K}{x}}$$

$$[\mathrm{P}]_0 = \mathrm{Z} \cdot [\mathrm{HA}]_0 \cdot \frac{\mathrm{Mw(charge)_{PDADMAC}}}{\mathrm{Mw(charge)}_{HA}}$$

Substitute the factors in remaining HA (%) $= \left(\frac{\mathrm{C}}{\mathrm{C_0}}\right) * 100\%$ to derive the theoretical remaining HA

$$\text{Theoretical remaining HA} = -\frac{\mathrm{Z} \cdot \mathrm{Mw(charge)_{PDADMAC}}}{2 \cdot \mathrm{x} \cdot \mathrm{Mw(charge)_{HA}}} + \frac{1}{2} + \sqrt{\left(\frac{\mathrm{Z} \cdot \frac{\mathrm{Mw(charge)_{PDADMAC}}}{\mathrm{Mw(charge)_{HA}}} - \mathrm{x}}{2 \cdot \mathrm{x}}\right)^2 + \frac{\mathrm{K}}{\mathrm{x} \cdot [\mathrm{HA}]_0{}^2}}$$

5. Static and Dynamic Light Scattering (SLS, DLS)

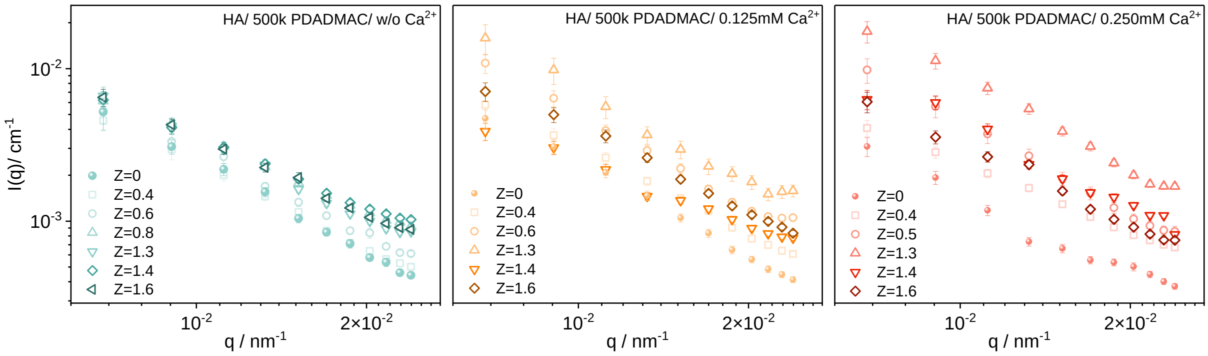

Figure S6. SLS intensities of HA-PDADMAC complexes under varying Ca^{2+} concentrations at different charge ratio Z at 25 °C.

Table S3. Characteristic stretching exponent α of HA-PDADMAC complexes under varying Ca^{2+} concentrations at different charge ratio Z at 25 °C.

Z	0	0.4	0.5	0.6	0.8	1.3	1.4	1.6
w/o Ca^{2+}	0.941	0.926		0.897	0.895	0.888	0.913	0.884
0.125mM Ca^{2+}	0.926	0.872		0.882		0.803	0.889	0.899
0.250mM Ca^{2+}	0.941	0.927	0.797			0.895	0.883	0.909

*determined from the DLS experiments with a stretched exponential fit

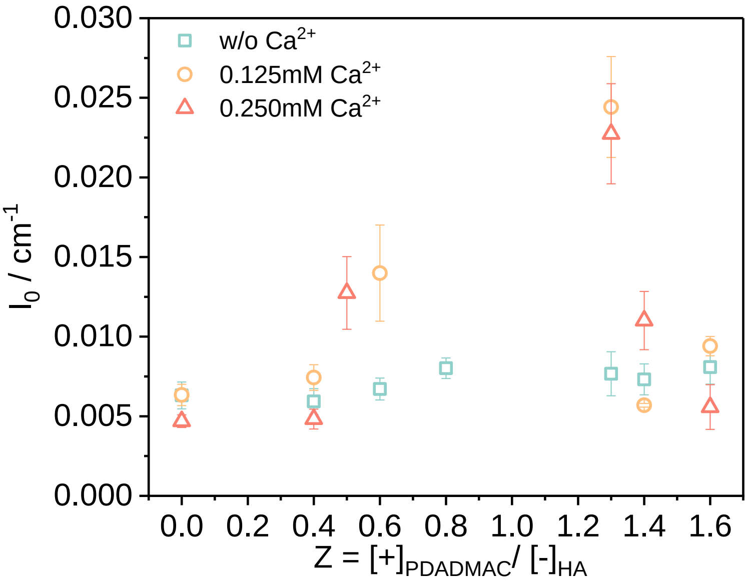

Figure S7. The static intensity at zero angle, I_0 under varying Ca^{2+} concentrations as a function of the charge ratio Z at 25 °C.

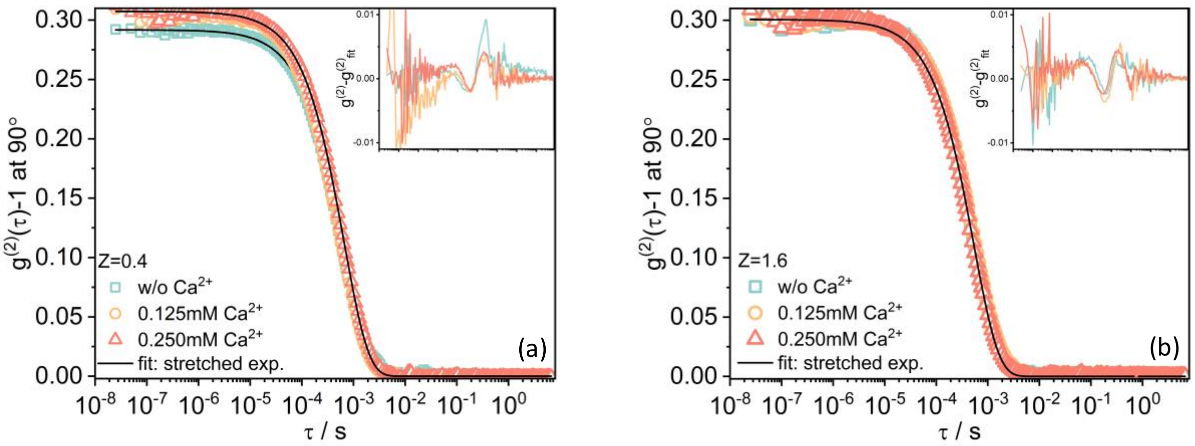

Figure S8. Intensity auto-correlation function $g^{(2)}(\tau)$ at 90° for HA-PDADMAC complexes at (a) Z =0.4 and (b) Z =1.6 under varying Ca^{2+} concentrations at 25 °C (c(HA) = 40 mg/L), as determined from the DLS experiments with a stretched exponential fit; The insets show the fit residuals, $g^{(2)}$-$g^{(2)}_{fit}$.

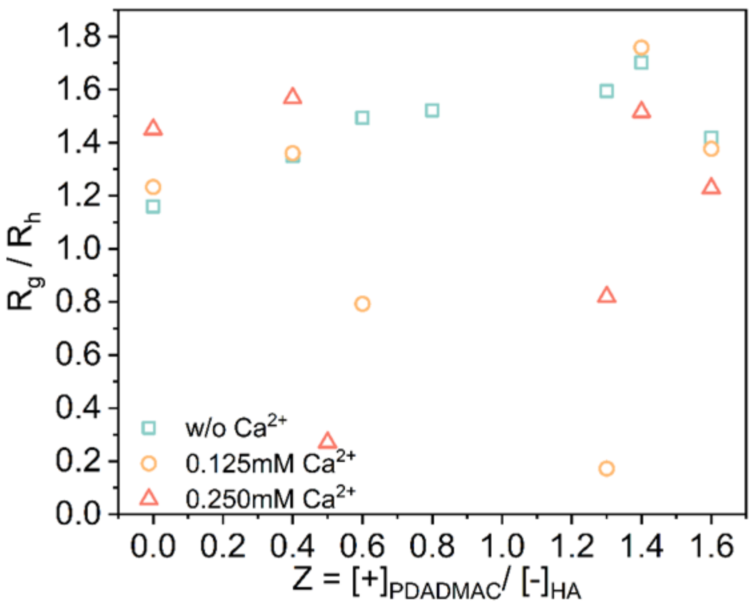

Figure S9. Ratio of radius of gyration and hydrodynamic radius Rg/Rh for HA-PDADMAC complexes under varying Ca^{2+} concentrations at different charge ratio Z at 25 °C

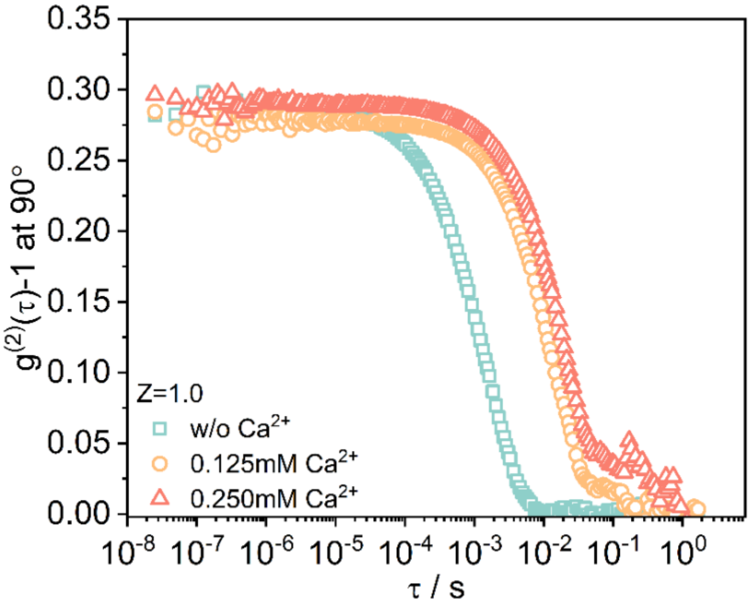

Figure S10. Intensity auto-correlation function at 90° for HA-PDADMAC complexes at Z =1.0 under varying Ca^{2+} concentrations (c(HA) = 40 mg/L). All the measurements were conducted 5 min after mixing.

6. Small-Angle Neutron Scattering (SANS)

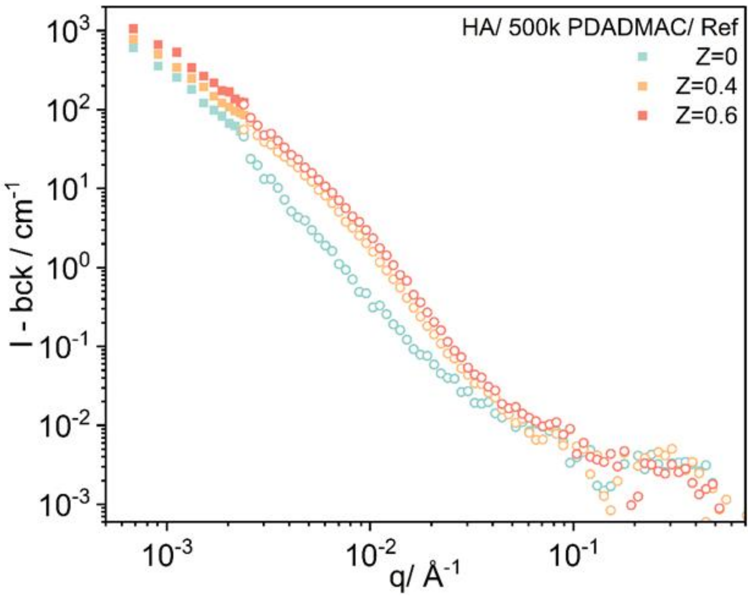

Figure S11. Combined SLS and SANS spectra for HA-PDADMAC complexes without added salt. To superimpose both techniques we plot converted the scattering intensity of SLS to the one of SANS by the corresponding contrast term, which is given by

$$I(q)SANS = I(q)SLS \frac{\lambda^4 \phi \Delta SLD^2}{4\pi^2 n^2 (\frac{dn}{dc})^2 c \rho_{agg}},$$

where λ is the wavelength of the incident light beam, ϕ is the volume fraction of all scattering particles, ΔSLD is the difference in scattering length density between particle and solvent, n is the refractive index of the solvent, (dn/dc) the refractive index increment, c is the mass concentration of solute and ρ_{agg} is the mean density of scattering particles.

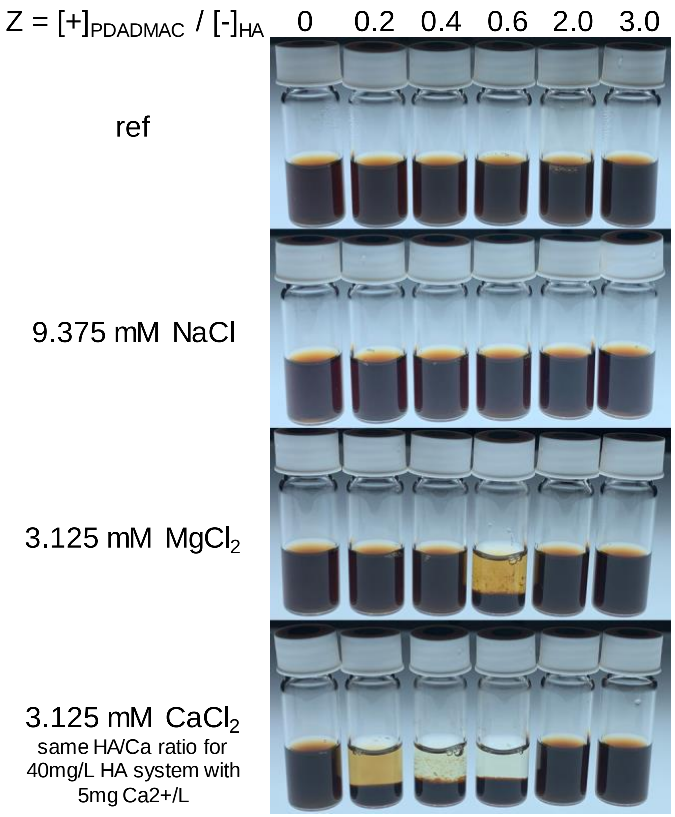

Figure S12. Photos of HA-PDADMAC complexes measured at ISIS with 1000 mg/L HA and different charge ratio Z values under different ionic conditions. Photos were taken after an aging time of 24 hours after mixing.

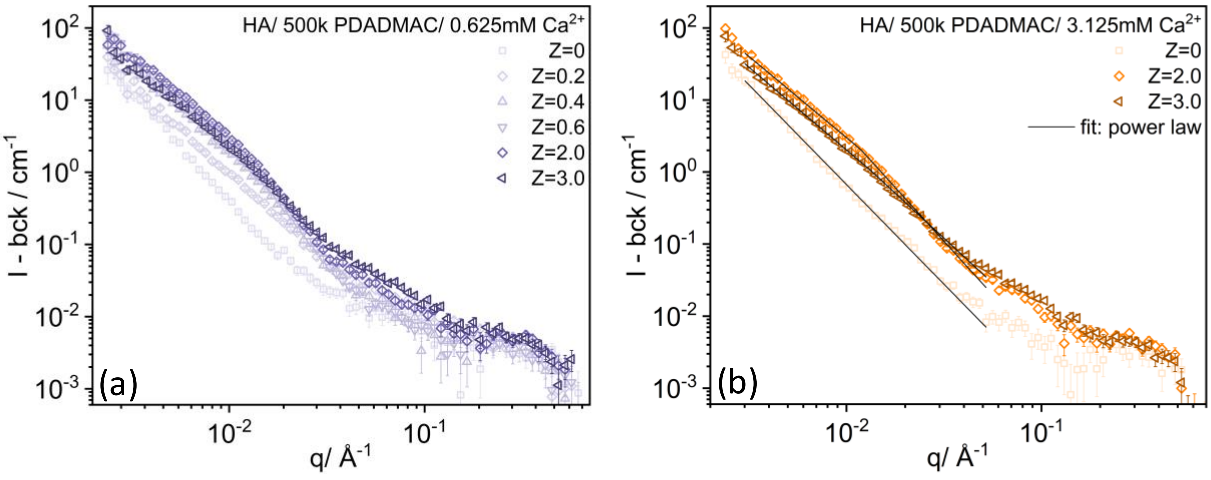

Figure S13. Scattering intensity as a function of the magnitude of the scattering vector q for HA-PDADMAC complexes with (a) 0.625 mM $CaCl_2$ and (b) 3.125 mM $CaCl_2$, respectively (c(HA) = 1.0 g/L).

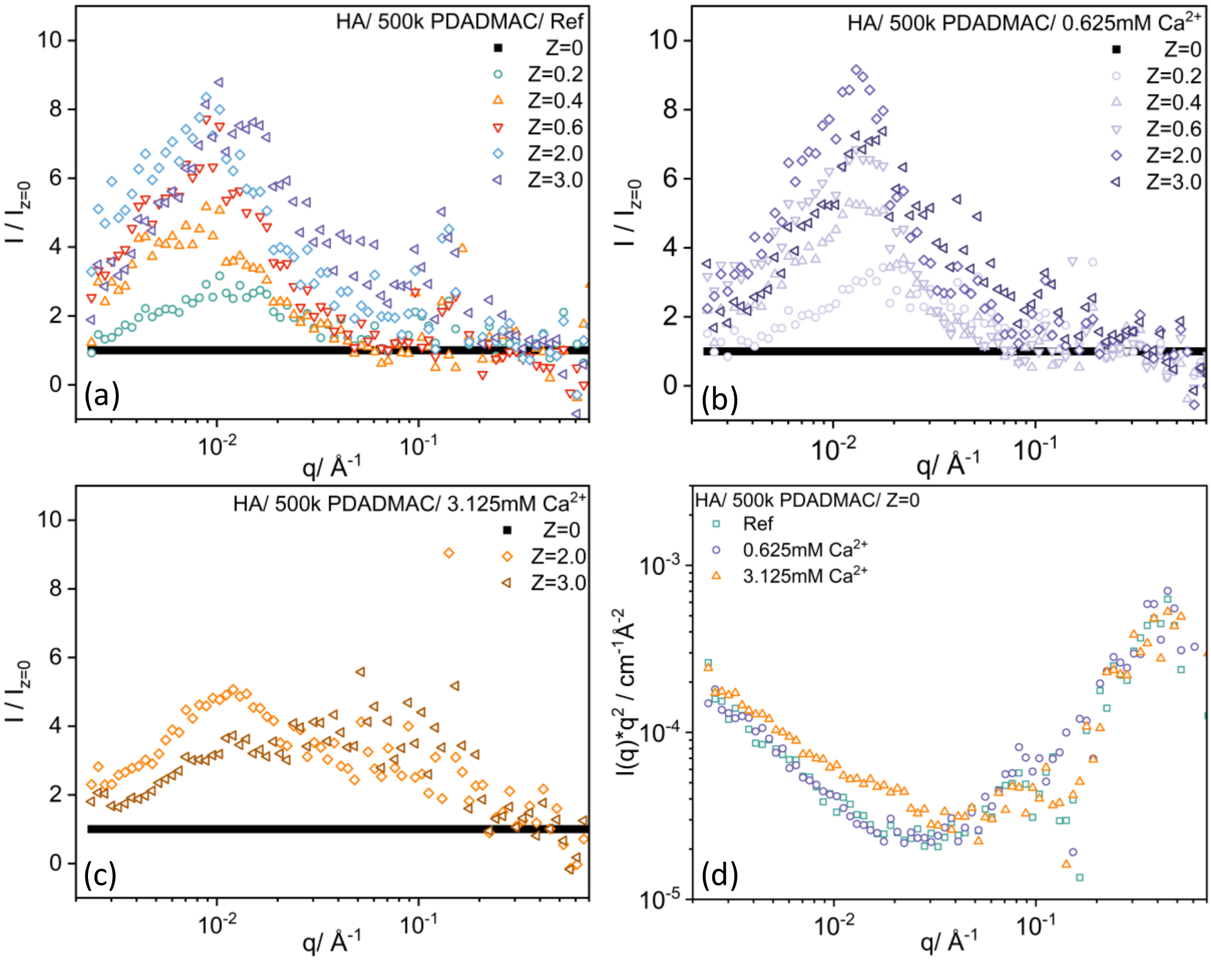

Figure S14. Scattering intensity as a function of the magnitude of the scattering vector q for HA-PDADMAC complexes without added salt (a), with 0.625 mM $CaCl_2$ (b), with 3.125 mM $CaCl_2$ (c) normalised to the respective pure HA intensity ($I/I_{Z=0}$) and the corresponding Kratky-Porod plot of the pure HA aggregates (Z = 0) (d).

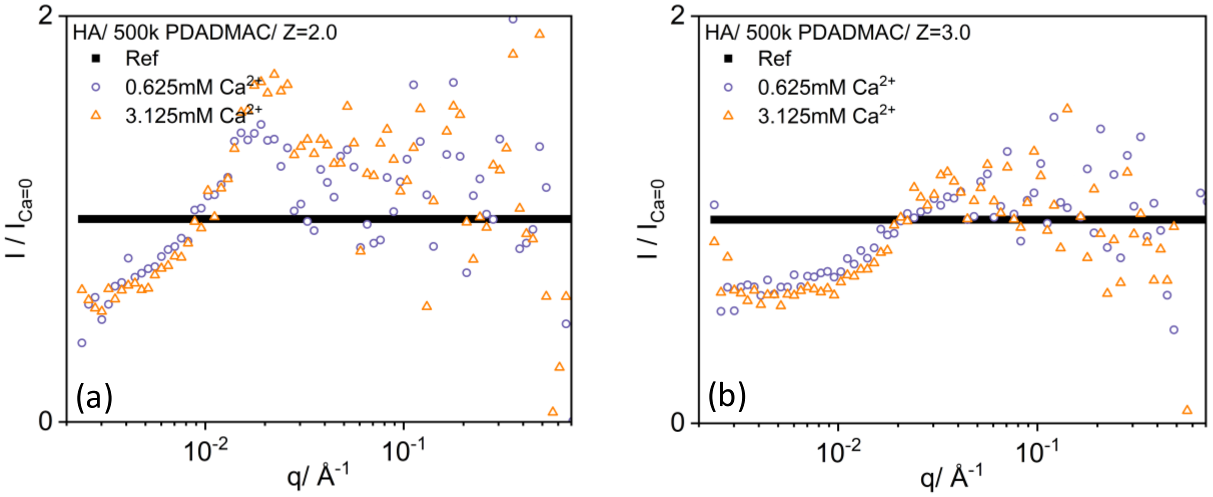

Figure S15. Scattering intensity as a function of the magnitude of the scattering vector q for HA-PDADMAC complexes for Z = 2.0 (a) and Z = 3.0 (b) for different addition of $CaCl_2$, normalised to the respective system without added $CaCl_2$.

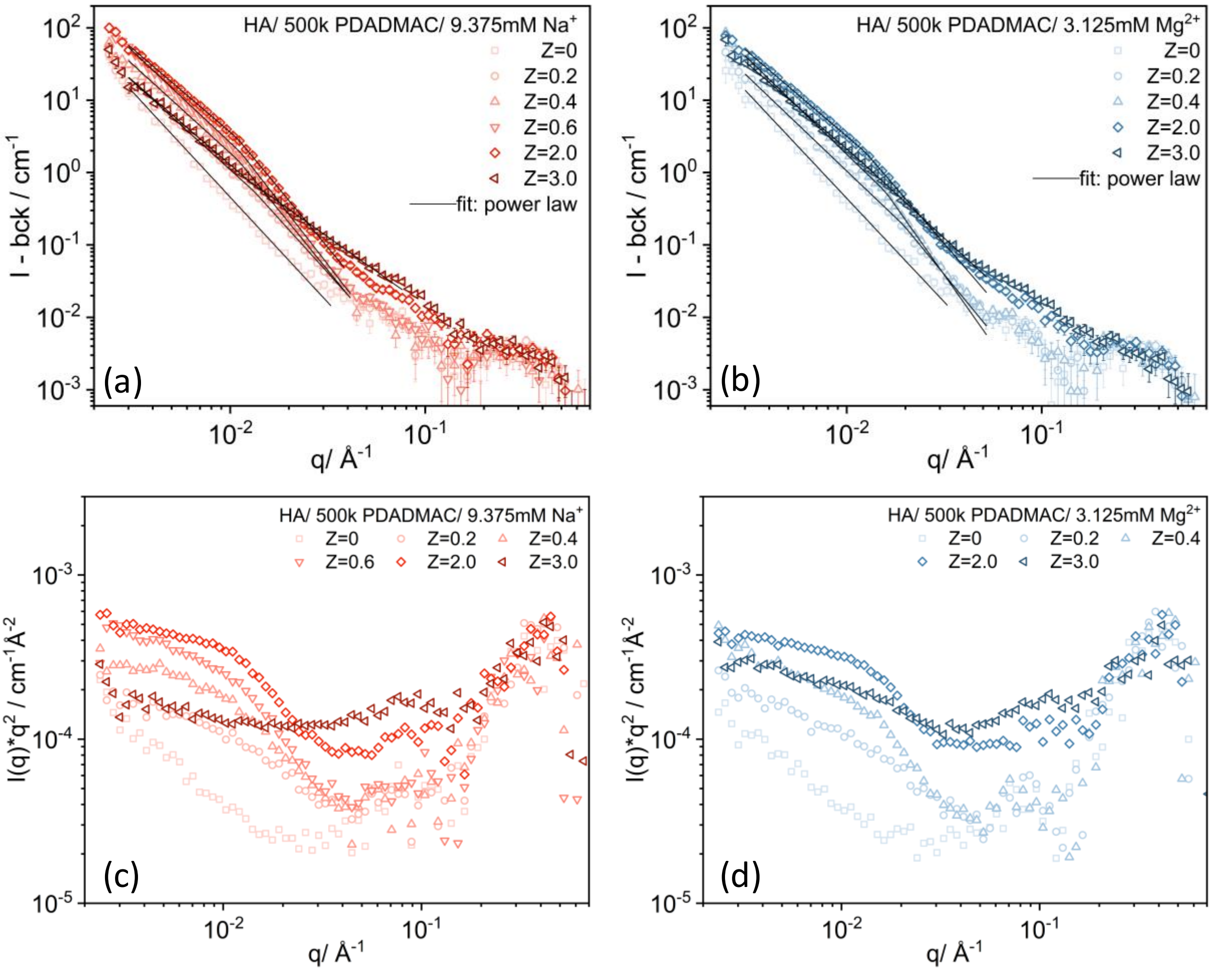

Figure S16. SANS intensity as a function of the magnitude of the scattering vector q for HA-PDADMAC complexes with addition of (a) 9.375 mM Na^+ and (b) 3.125 mM Mg^{2+}, respectively, and the corresponding Kratky-Porod plots (c(HA) = 1.0 g/L) (c) and (d).

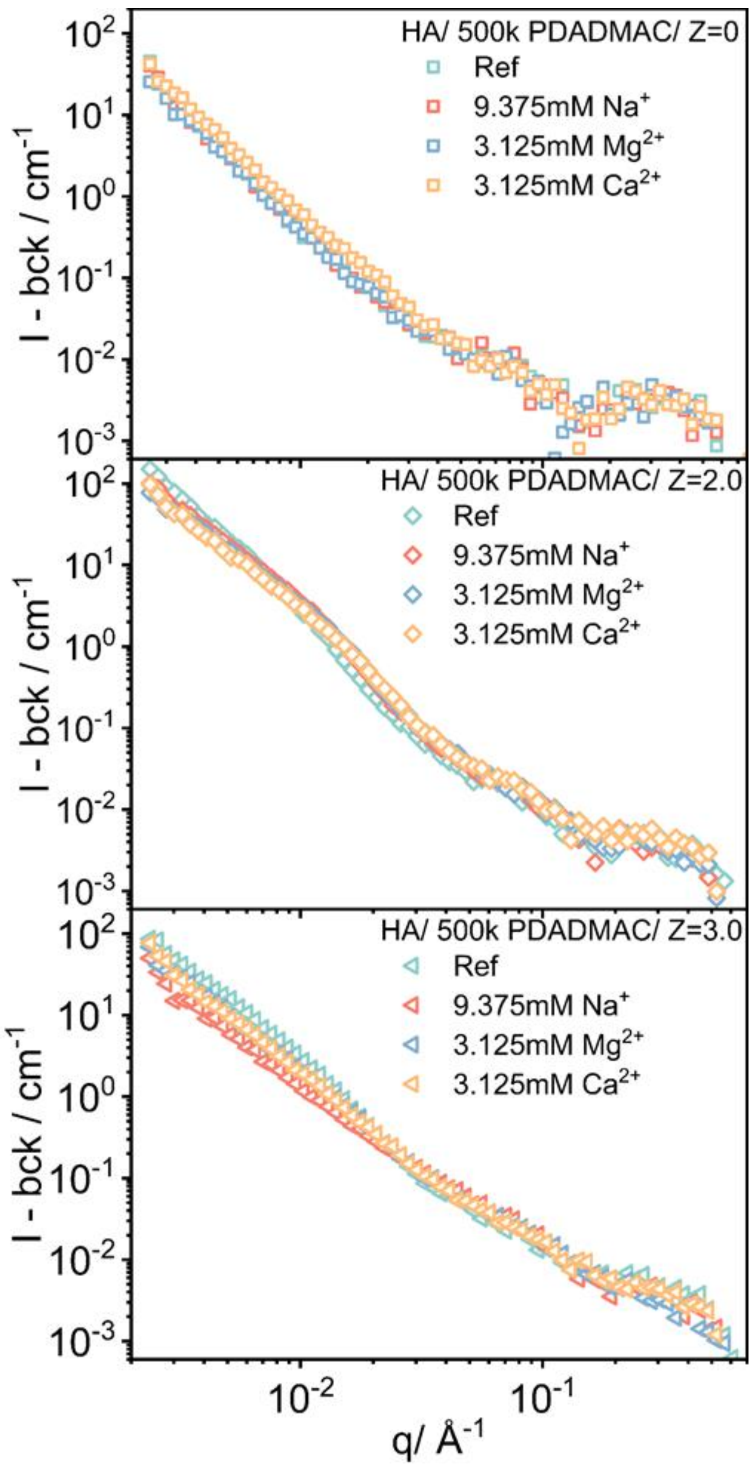

Figure S17. SANS intensity as a function of the magnitude of the scattering vector q for pure HA (Z = 0) under various ionic conditions and for HA-PDADMAC complexes under various ionic conditions at Z = 2 and 3, respectively (c(HA) = 1.0 g/L).

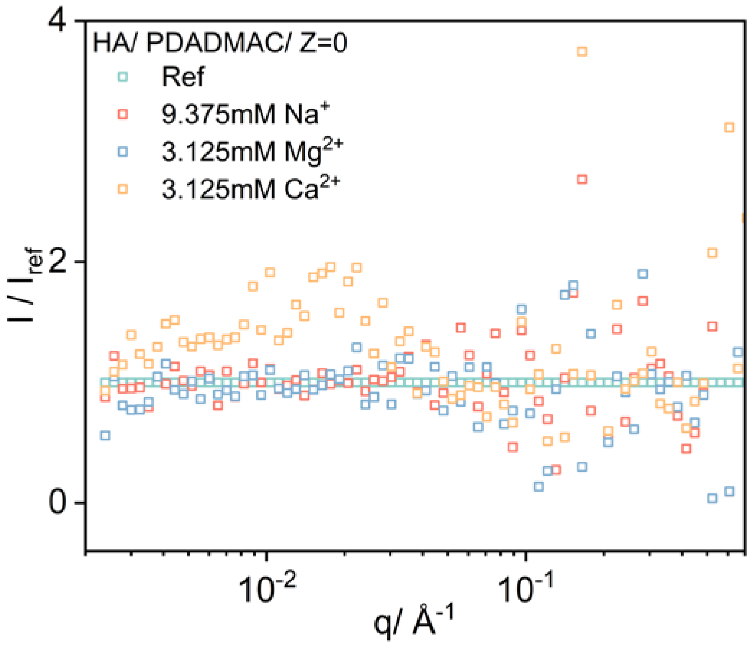

Figure S18. Scattering intensity as a function of the magnitude of the scattering vector q for HA complexes (c(HA) = 1.0 g/L) at Z = 0 under various ionic conditions normalized to the intensity of pure HA without added salt (I/I_{Ref}).

	Z	R_{g1}, nm	P_1	B_1, 10^{-6}cm^{-1}nm^{-p}	G_1, cm^{-1}	R_{g2}, nm	P_2	B_2, 10^{-3}cm^{-1}nm^{-p}	G_2, cm^{-1}
**ref**	**0**	43.7	2.06	2.37	15.6				
	**0.2**	56.1	2.79	2.96	51.2	5.19	0.78	1.31	0.03
	**0.4**	58.2	3.44	2.33	95.8	5.51	0.46	1.52	0.01
	**0.6**	62.4	2.91	4.41	154	7.68	1.24	0.42	0.11
	**2**	76.8	2.85	6.83	331	9.01	1.36	0.52	0.25
	**3**	95.5	2.31	8.18	341	9.60	1.45	0.57	0.49
**Na**	**0**	43.0	1.98	3.27	14.9				
	**0.2**	60.8	2.77	3.12	50.1	2.34	0.22	1.97	0.01
	**0.4**	53.6	2.79	5.26	65.2	6.24	0.99	0.63	0.06
	**0.6**	63.4	2.57	2.15	143	8.39	1.25	0.42	0.14
	**2**	64	2.5	1.40	156	10.2	1.59	3.10	0.52
	**3**	52.9	2.74	3.72	38.5	8.05	1.77	0.30	0.48
**Mg**	**0**	44.4	2.14	1.72	16.2				
	**0.2**	58.6	2.59	7.49	53.9	4.45	0.56	1.46	0.02
	**0.4**	108	2.40	3.29	508	7.47	0.82	0.96	0.06
	**2**	56.3	3.55	1.54	94.1	13.7	1.73	0.23	3.80
	**3**	59.4	2.28	6.17	84.8	8.25	1.63	0.39	0.40
**Ca**	**0**	56.1	2.49	6.38	41.9				
	**2**	86.8	2.12	1.42	278	8.30	1.37	0.61	0.30
	**3**	102	2.30	0.52	324	7.41	1.55	0.48	0.31

Table S4. Radius of gyration at larger scale R_{g1} and smaller scale R_{g2} as well as the corresponding scaling pre-factor G, power law scattering pre-factor B and power law exponent P as determined by the Beaucage model from the SANS data of HA complexes with different charge ratio Z values under different ionic conditions.

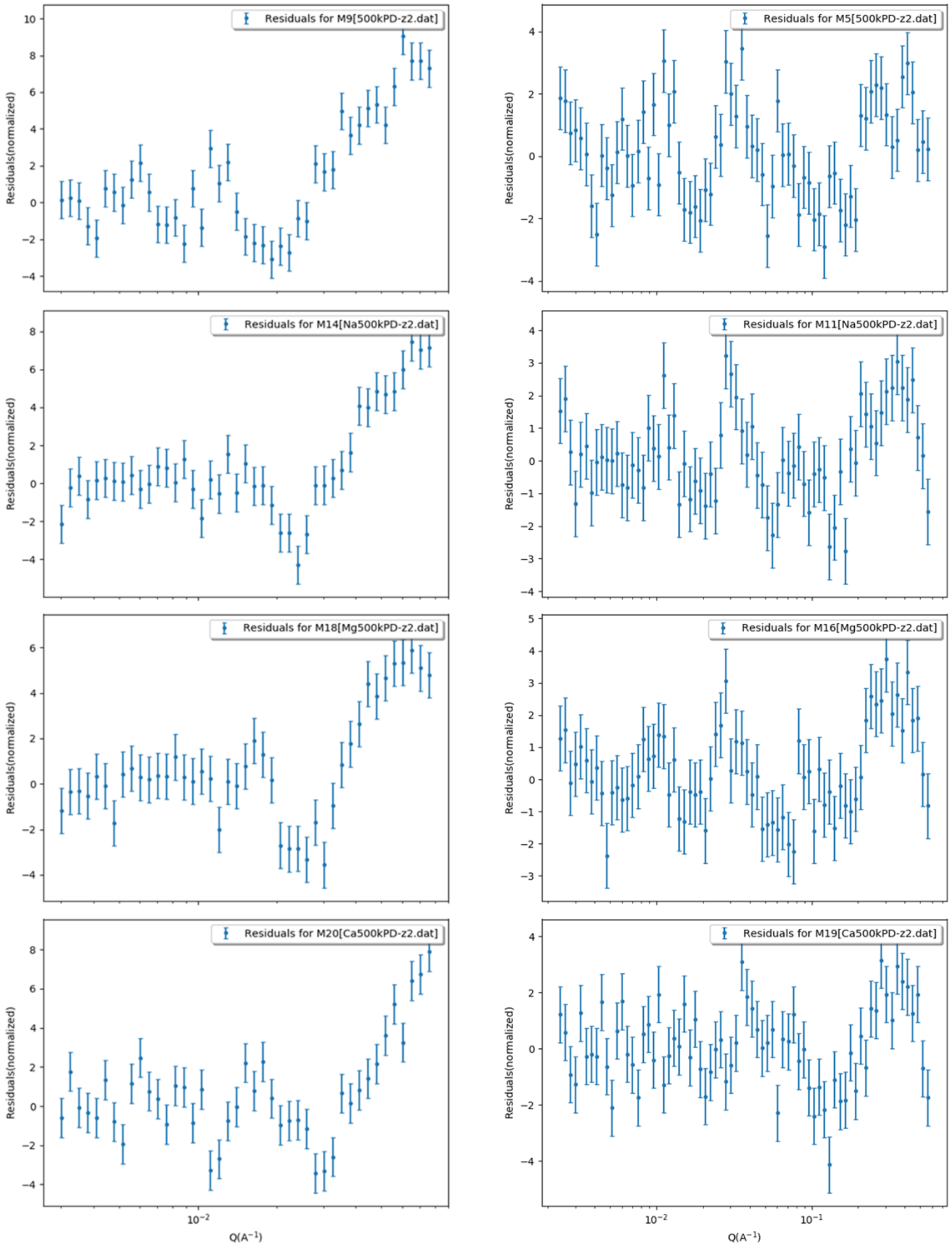

Figure S19. Corresponding residuals for fit of SANS data for 500kDa PDADMAC-HA complexes under various ionic conditions at Z = 2 with the two-power law model (left column) and Beaucage model (right column).

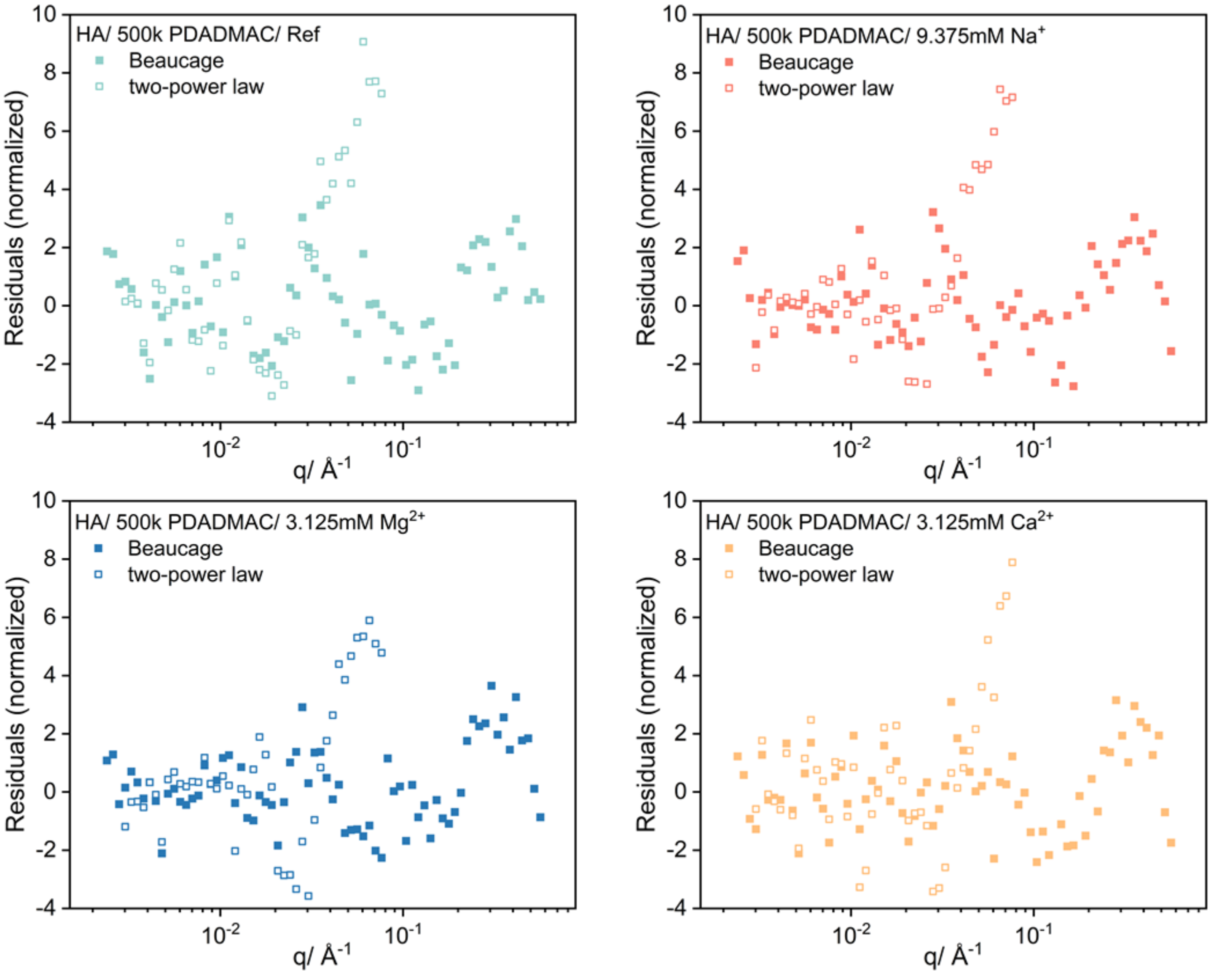

Figure S20. Corresponding residuals for fit of SANS data for 500kDa PDADMAC-HA complexes under various ionic conditions at Z = 2 with the two-power law model and Beaucage model.
